# Effect of methylphenidate on physical growth indicators in children and adolescents with attention-deficit/hyperactivity disorder: a systematic review and meta-analysis

**DOI:** 10.3389/fpsyt.2026.1794403

**Published:** 2026-05-11

**Authors:** Minxin Cao, Tao Song, Xiaoli Hou, Xiaohui Yu

**Affiliations:** 1 Zhongshan Clinical College, Dalian University, Dalian, Liaoning, China; 2 Pediatrics, Affiliated Zhongshan Hospital of Dalian University, Dalian, Liaoning, China

**Keywords:** ADHD, growth, meta-analysis, methylphenidate, stimulant

## Abstract

**Background:**

Methylphenidate (MPH) is the first-line medication for attention deficit hyperactivity disorder (ADHD). The results regarding its potential negative impact on the physical growth of children and adolescents with ADHD are inconsistent. Accordingly, the present research aimed to systematically evaluate the influence of MPH monotherapy on physical growth parameters in children and adolescents with ADHD.

**Methods:**

A systematic search was conducted across the Cochrane Library, Embase, PubMed, and Web of Science up to December 2025 to identify clinical studies reporting pre- and post-treatment measurements of physical growth parameters—including height, weight, and body mass index (BMI)—in children and adolescents with ADHD receiving MPH monotherapy. EndNote was employed for literature screening. Eligible studies underwent data extraction and quality evaluation. Cohort studies were evaluated utilizing the Newcastle-Ottawa Scale, whereas randomized controlled trials were assessed with the RoB 2.0. The certainty of evidence was assessed using the GRADE approach. Statistical analyses were implemented in STATA 18.0.

**Results:**

Thirty-three studies were incorporated, of which 16 were rated as high quality and 17 as moderate quality. Meta-analysis indicated that MPH-treated children with ADHD exhibited significant reductions in height Z-score (mean difference [MD] = -0.13, 95% confidence interval [CI] = -0.18 to -0.09; I² = 91.1%, p < 0.001), weight Z-score (MD = -0.25, 95% CI = -0.36 to -0.15; I² = 96.8%, p < 0.001), short-term mean weight (MD = -0.34, 95% CI = -0.51 to -0.18; I² = 0.0%, p = 0.449), and body mass index Z-score (MD = -0.26, 95% CI = -0.32 to -0.20; I² = 96.3%, p < 0.001), while mean BMI showed no statistically significant difference (MD = 0.20, 95% CI = -0.24 to 0.64; I² = 96.0%, p = 0.449). For outcomes with substantial heterogeneity, subgroup and regression analyses were conducted, but no clear sources were identified.

**Conclusions:**

MPH use is significantly associated with reductions in growth parameters in children and adolescents with ADHD. Even though the effect sizes were small, clinicians should prioritize individualized monitoring. Future research should employ more standardized prospective designs to minimize potential confounders.

**Systematic review registration:**

https://www.crd.york.ac.uk/PROSPERO/view/CRD420251117485, identifier CRD420251117485.

## 1 Introduction

Attention deficit hyperactivity disorder (ADHD) represents a prevalent chronic neurodevelopmental condition marked by inattention, hyperactivity, and impulsivity. Its prevalence exhibits a rising trend worldwide, with an estimated incidence of approximately 7.2% among children (1). Notably, symptoms persist into adulthood for some individuals (2), and the disorder demonstrates a higher prevalence in males. Children and adolescents with ADHD face greater challenges in the developmental trajectory than their peers who develop typically. This exerts adverse impacts across multiple life domains. These encompass compromised academic performance, social interactions, family relationships, and an elevated risk for a broad spectrum of psychiatric comorbidities (3). Difficulties in managing uncontrolled impulsive behaviors and inattention often manifest as classroom disruption and lower academic achievement, and an increased likelihood of school dropout is seen in severe cases (4, 5). Furthermore, impulsivity elevates risks for accidental injuries and exposure to toxic substances (6).

For school-aged children, pharmacotherapy constitutes the first-line intervention. Cortese et al.’s retrospective review indicates that early effective treatment correlates with more favorable prognoses and fewer problems in adulthood (7). Methylphenidate (MPH), a central nervous system stimulant, serves as a primary pharmacological agent. Its mechanism of action involves inhibiting the activity of synaptic transporter proteins (8), thereby reducing the reuptake of norepinephrine and dopamine by presynaptic neurons. This process enhances the functional efficiency of the prefrontal cortex and improves noradrenergic and dopaminergic modulation of subcortical and cortical circuits. These neurochemical enhancements ultimately ameliorate attentional and executive functions in individuals with ADHD (9, 10). However, persistent concerns exist among parents and researchers regarding the potential negative impacts of MPH on child growth and development. Current evidence remains inconsistent on whether ADHD pharmacotherapy affects physical growth in children (11–13). Several mechanisms may underlie the potential influence of MPH on growth. Firstly, diminished appetite constitutes a common adverse effect, and insufficient nutritional intake to meet developmental demands likely constitutes a direct factor. Endogenous peptides might also contribute notably to the neurobiological mechanisms of MPH-associated anorectic effects (14). Concurrently, MPH can negatively affect sleep and directly increase dopaminergic activity, potentially suppressing growth hormone secretion. Additional proposed mechanisms comprise deceleration of cartilage tissue growth or modulation of osteoclast activity (15–21).

Existing meta-analyses primarily focus on the influence of MPH treatment on height within this specific population. Our meta-analysis aims to delineate the overall effect and clinical significance of MPH on children and adolescents with ADHD in terms of weight, body mass index (BMI), and height. This work might assist clinicians in implementing targeted monitoring and personalized interventions to balance treatment efficacy with safety.

## 2 Materials and methods

The present meta-analysis was implemented per the Preferred Reporting Items for Systematic Reviews and Meta-Analyses guidelines (22) (**Supplementary Material 1**). Its protocol has been prospectively registered with the International Prospective Register of Systematic Reviews database (CRD420251117485).

### 2.1 Eligibility criteria

Literature meeting the following criteria was eligible for inclusion: (i) Subjects: Children and adolescents diagnosed with ADHD per DSM-III/IV/V or ICD-10 criteria, aged <18 years; (ii) Intervention: MPH monotherapy, with a minimum treatment duration of three weeks; (iii) Primary outcomes: Reported changes in growth parameters measured before and after treatment within the intervention group, including height [standard deviation score (SDS) or Z-score], weight (measured value, SDS, or Z-score), and BMI (measured value, SDS, or Z-score); (iv) Study design: Prospective or retrospective observational studies, and randomized controlled trials (RCTs).

Exclusion criteria comprised: (i) Animal experiments, reviews, case reports, editorials, conference abstracts, etc.; (ii) Non-English publications; (iii) Trials that did not specify the stimulant medication or involved medication switches during treatment; (iv) Concurrent use of other psychotropic medications during the treatment period; (v) Studies where full texts were unavailable despite contact attempts, or where data were missing or contained serious errors.

### 2.2 Search strategy

A comprehensive literature search was conducted across the Cochrane Library, Embase, PubMed, and Web of Science up to December 16, 2025. The search strategy combined Medical Subject Headings with free-text words related to ADHD, MPH, and growth parameters, including “ADHD,” “methylphenidate,” “height,” “weight,” and “BMI.” **Supplementary Material 2** provides the complete search strategy for each database. Reference lists of included studies, along with those of relevant systematic reviews, were scrutinized to supplement the search and ensure comprehensiveness.

### 2.3 Literature screening and data collection

EndNote was applied to import retrieved records. Two independent investigators reviewed titles and abstracts against the eligibility criteria. A full-text review was then conducted for secondary screening. Discrepancies were resolved via discussion or consultation with a third investigator. Two investigators independently collected information from eligible studies employing Excel 2016. The information encompassed author, publication year, title, study design, country, details of subjects (mean age, sex, sample size), diagnostic criteria, MPH treatment specifics (formulation, dose, duration), and outcome measures.

### 2.4 Quality evaluation

Two investigators appraised the quality of cohort studies employing the Newcastle-Ottawa Scale (NOS) (23), evaluating patient selection, outcome assessment, and group comparability. The NOS awards a maximum of nine stars; studies with ≥ 6 stars were deemed high quality. The Cochrane Risk of Bias tool (RoB 2.0) was applied (24) for RCTs to examine bias from the randomization process, outcome measurement, missing outcome data, selective reporting, and deviations from intended interventions. High risk, some concerns, or low risk was assigned as the rating for each domain. Discrepancies were settled through team discussion or by consulting a third investigator. Results were presented in a risk of bias (RoB) graph.

The certainty of evidence for each primary outcome was independently examined by two reviewers using the Grading of Recommendations Assessment, Development and Evaluation (GRADE) framework. Evidence derived from RCTs initially received a rating of high certainty, which was subsequently downgraded based on five domains: risk of bias, inconsistency, indirectness, imprecision, and publication bias. Evidence from observational studies could be upgraded based on considerations such as a large magnitude of effect, the presence of a dose–response gradient, or when unmeasured confounding factors were likely to cause underestimated true effects—that is, where adjustment for residual confounding would have strengthened rather than attenuated the observed association. The final rating of certainty was classified into four levels: high, moderate, low, or very low.

### 2.5 Statistical analysis

Statistical analyses were implemented in STATA (v18.0). All data were continuous variables. Mean differences (MDs) served as the pooled effect measure, with 95% confidence intervals (CIs) reported. Inter-study heterogeneity was evaluated utilizing the I² statistic and Q-test. An I² ≥ 50% and a P-value < 0.10 indicated substantial heterogeneity, prompting the use of a random-effects model; otherwise, a fixed-effects model was applied. Subgroup analysis and meta-regression were implemented to investigate possible sources of heterogeneity. Leave-one-out sensitivity analyses were carried out to evaluate result stability. Publication bias was appraised via funnel plots and Egger’s test. Any notable bias was adjusted using the trim-and-fill method.

## 3 Results

### 3.1 Literature screening

**Figure 1:**
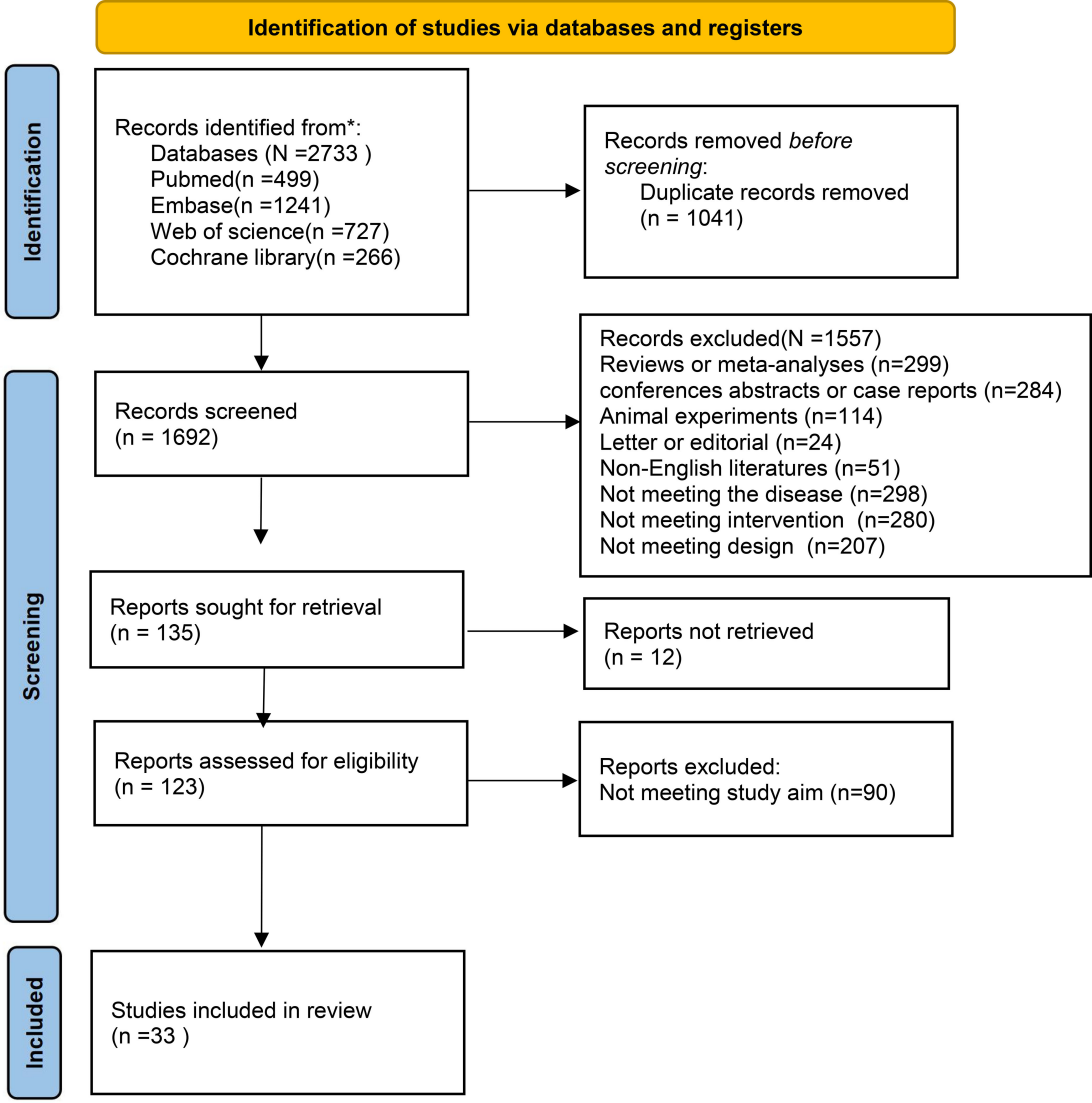
Literature screening flowchart.

The initial database retrieval yielded 2,733 records. After removing 1,041 duplicates, 1,692 unique records underwent title/abstract screening, and 1,569 items (e.g., reviews, conference abstracts, animal experiments, case reports) were removed. The remaining 123 studies were reviewed in full text, and 90 studies lacking valid data were excluded. Ultimately, 33 studies were encompassed in the meta-analysis (14, 17, 25–55). **Figure 1** illustrates the literature screening process.

### 3.2 Characteristics and RoB of eligible studies

The 33 eligible investigations involved 4,438 subjects, with a predominance of males (five studies enrolled males exclusively). Ages ranged from three to 18 years, with two studies focusing solely on preschoolers (three to six years). MPH treatment duration varied from three weeks to five years. Oral MPH included immediate- or extended-release formulations, with most studies involving multiple formulations. Among the 33 studies, four were RCTs, 15 were prospective, and 14 were retrospective. The interventions in the control group varied in the four RCTs, consisting of placebo, drug holidays, MPH combined with risperidone, or dexmethylphenidate. Consequently, the quantitative synthesis was limited to analyzing pre- and post-intervention data within the group of MPH treatment (**Supplementary Material 3**).

**Figure 2:**
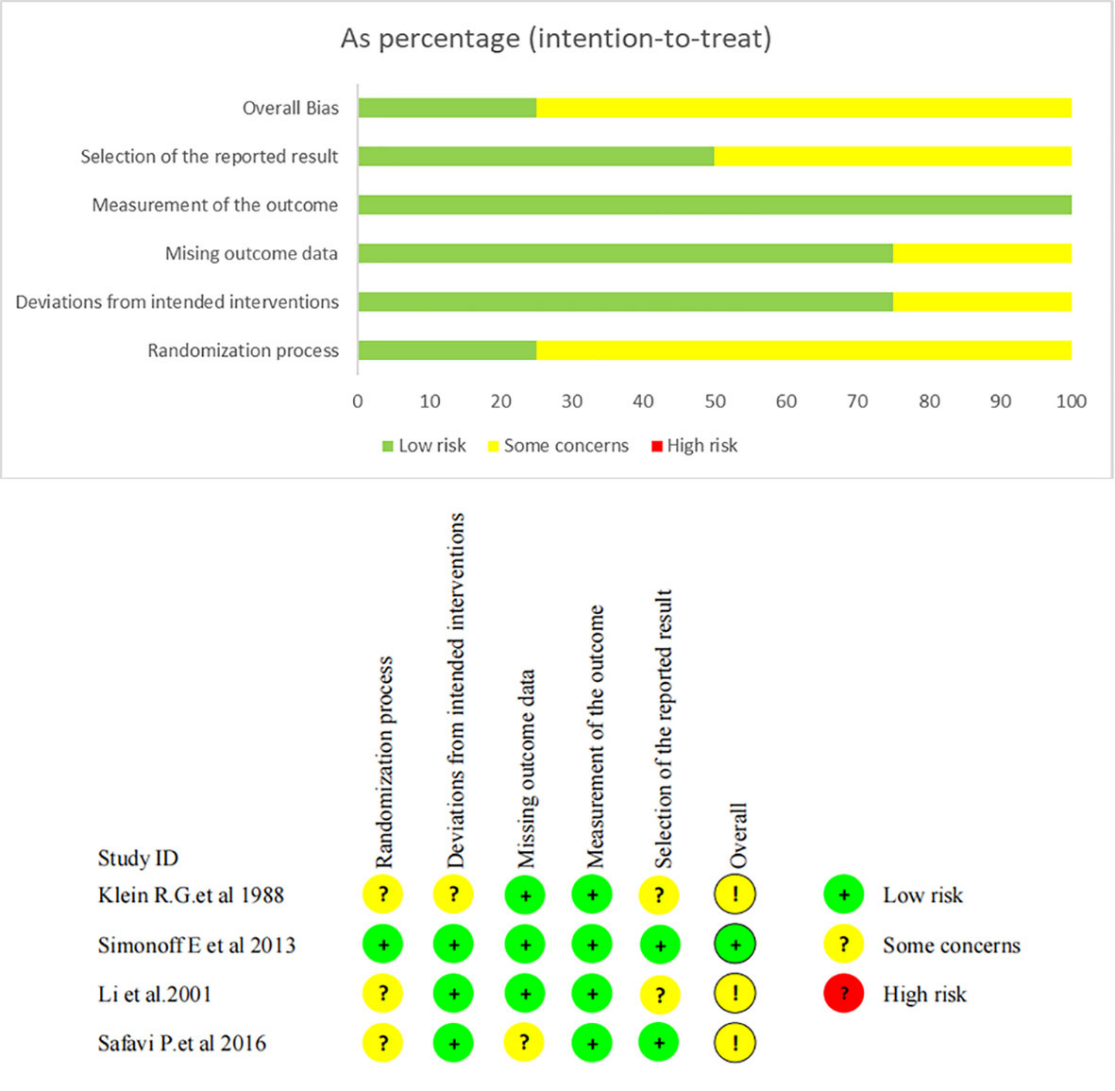
The quality assessment of RCTs.

The quality of the 29 cohort studies was assessed, with 15 rated as high and 14 as medium quality (**Supplementary Material 4**). For the four eligible RCTs, risk-of-bias evaluation using RoB 2.0 is summarized in **Figure 2**. Within the domain of bias arising from the randomization process, one study was judged to be at low risk, while three were deemed to have some concerns due to unclear allocation concealment. Regarding bias due to missing outcome data, three investigations were at low risk, and one raised some concerns because of participant attrition. Overall, the eligible studies demonstrated a generally low RoB.

### 3.3 GRADE assessment

Owing to heterogeneity across studies in the design of the control group and reporting of outcomes, the number of studies available for specific comparisons of each outcome was relatively limited. Following the assessment of GRADE, the certainty of evidence for most outcomes was rated as low. Evidence for height Z-score (HZS) was downgraded to very low due to considerable inconsistency across studies (high I²) and imprecision. Evidence for the remaining outcomes was also downgraded due to limited sample sizes or imprecision in effect estimates (**Supplementary Material 5**).

### 3.4 Meta-analysis results

#### 3.4.1 Height

**Figure 3:**
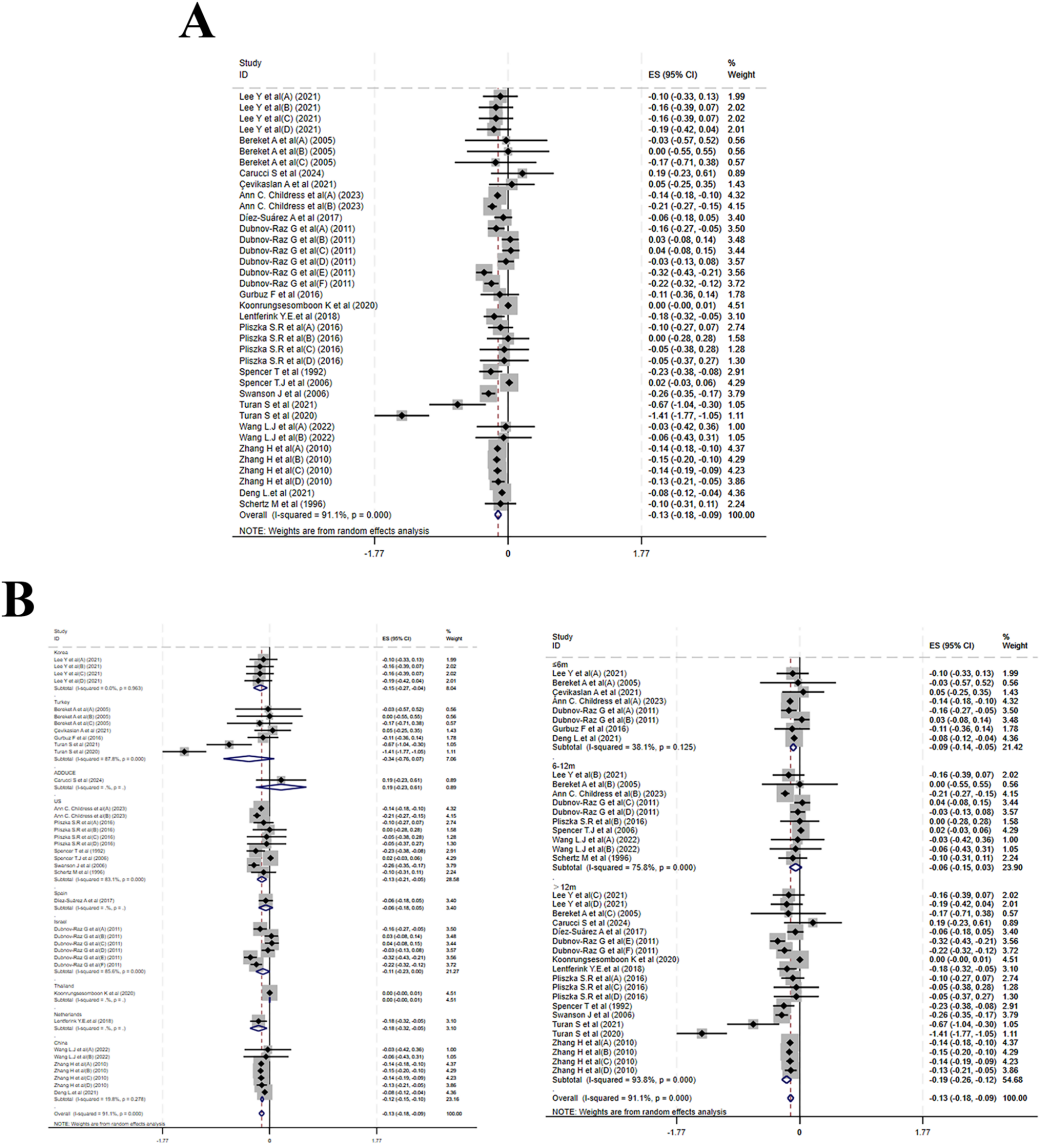
Meta-analysis results of height z-scores. **(A)** Forest plot of height z-scores; **(B)** Subgroup analysis of height z-scores by country and follow-up time.

Twenty studies reported changes in HZS. MPH treatment led to a marked reduction in HZS (MD = -0.13, 95% CI: -0.18 to -0.09; I² = 91.1%, p < 0.001), indicating a high degree of heterogeneity(**Figure 3A**). Subgroup analysis by treatment duration yielded MDs of -0.09 (-0.14 to -0.05; I² = 38.1%, P = 0.125) for ≤ 6 months, -0.06 (-0.15 to 0.03; I² = 75.8%, p < 0.001) for 6–12 months, and -0.19 (-0.26 to -0.12; I² = 93.8%, p < 0.001) for > 12 months. Country-stratified results were as follows: a pooled MD of –0.13 (–0.21 to –0.06; I² = 83.1%, p < 0.001) from six studies in the USA; –0.34 (–0.76 to 0.07; I² = 87.8%, p < 0.001) from five studies in Turkey; and –0.12 (–0.15 to –0.10; I² = 19.8%, P = 0.278) from three studies in China. Single-study estimates were –0.15 (–0.27 to –0.04) from Korea, –0.06 (–0.18 to 0.05) from Spain, –0.11 (–0.23 to 0.00) from Israel, 0.00 (–0.00 to 0.01) from Thailand, –0.18 (–0.32 to –0.05) from the Netherlands, and 0.19 (–0.23 to 0.61) from one multicenter study (**Figure 3B**). Meta-regression of baseline age, sample size, and publication year identified no significant source of heterogeneity (**Supplementary Material 6.1**).

#### 3.4.2 Weight

**Figure 4:**
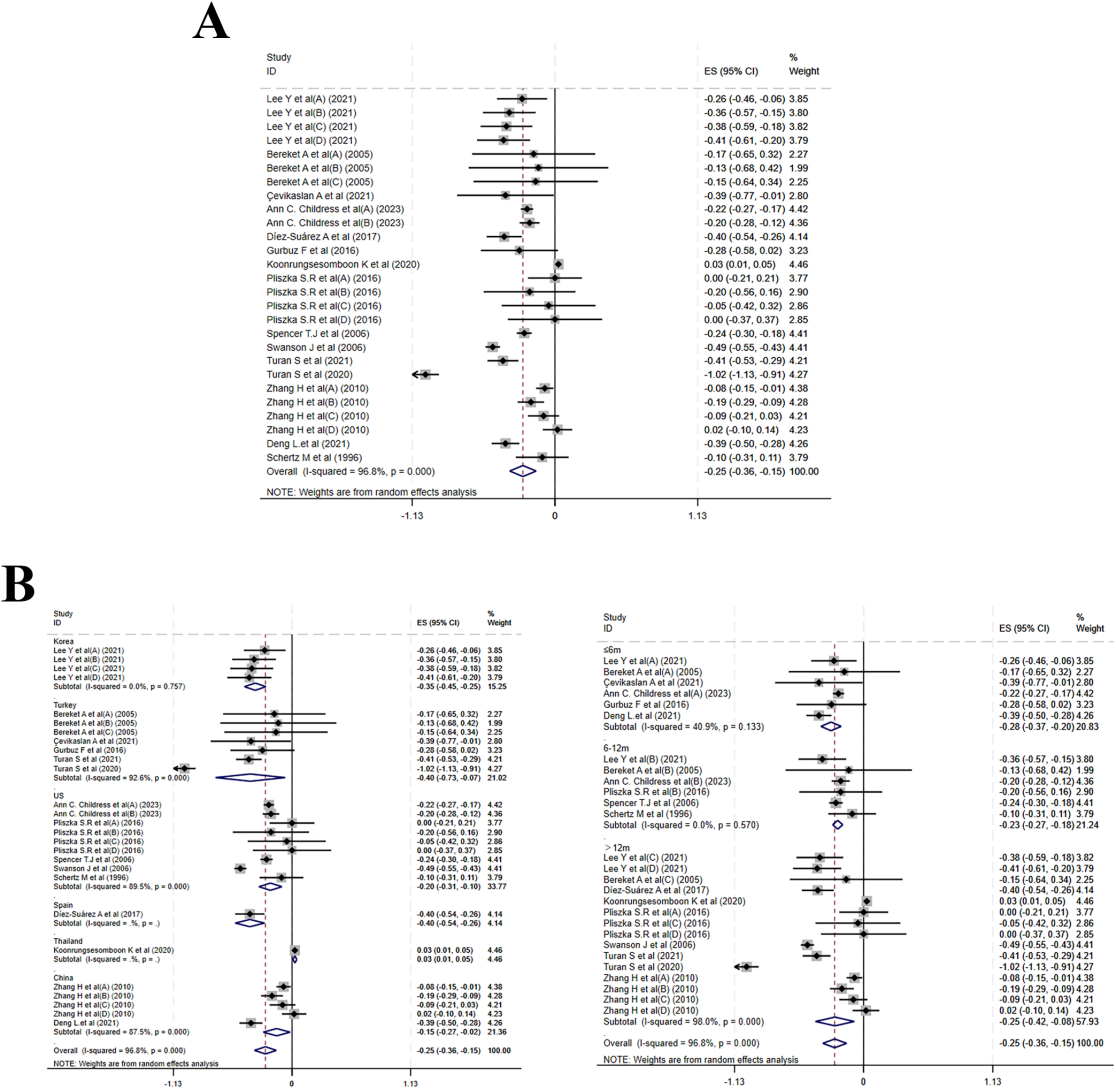
Meta-analysis results of weight z-scores. **(A)** Forest plot of weight z-scores; **(B)** Subgroup analysis of weight z-scores by country and follow-up time.

Fifteen studies reported changes in weight Z-score (WZS). Compared to baseline, MPH resulted in a notable reduction in WZS (MD = –0.25, 95% CI: –0.36 to –0.15; I² = 96.8%, p < 0.001)(**Figure 4A**). Subgroup analysis by treatment duration indicated MDs of –0.28 (–0.37 to –0.20; I² = 40.9%, P = 0.133) for ≤ 6 months, –0.23 (–0.27 to –0.18; I² = 0.0%, p = 0.570) for 6–12 months, and –0.25 (–0.42 to –0.08; I² = 98.0%, p < 0.001) for >12 months. Country-stratified analysis yielded the following estimates: a pooled MD of –0.20 (–0.31 to –0.10; I² = 89.5%, p < 0.001) from five studies in the USA; –0.40 (–0.73 to –0.07; I² = 92.6%, p < 0.001) from five studies in Turkey; –0.15 (–0.27 to –0.02; I² = 87.5%, p < 0.001) from two studies in China; –0.35 (–0.45 to –0.25) from one study in Korea; –0.40 (–0.54 to –0.26) from one study in Spain; and 0.03 (0.01 to 0.05) from one study in Thailand (**Figure 4B**). Meta-regression of baseline age, sample size, and publication year identified no significant source of heterogeneity (**Supplementary Material 6.2**).

**Figure 5:**
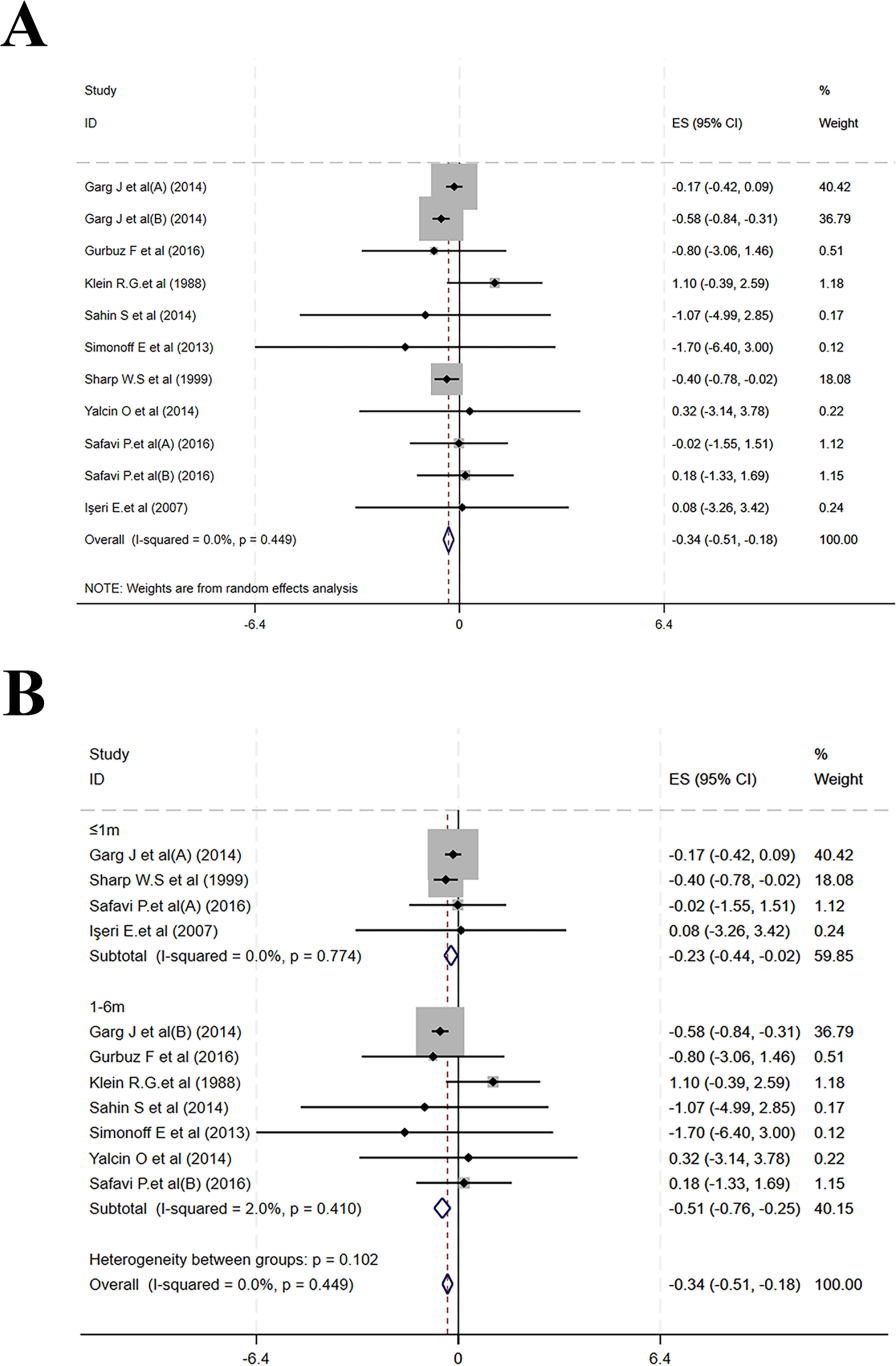
Meta-analysis results of mean weight. **(A)** Forest plot of mean weight; **(B)** Subgroup analysis of mean weight by follow-up time.

Nine studies reported changes in mean weight within six months of MPH treatment. Post-treatment mean weight was notably reduced (MD = –0.34, 95% CI: –0.51 to –0.18; I² = 0.0%, p = 0.449)(**Figure 5A**). Subgroup analysis yielded MDs of –0.23 (–0.44 to –0.02; I² = 0.0%, P = 0.774) for ≤ 1 month and –0.51 (–0.76 to –0.25; I² = 2.0%, p = 0.410) for 1–6 months (**Figure 5B**).

#### 3.4.3 BMI

**Figure 6:**
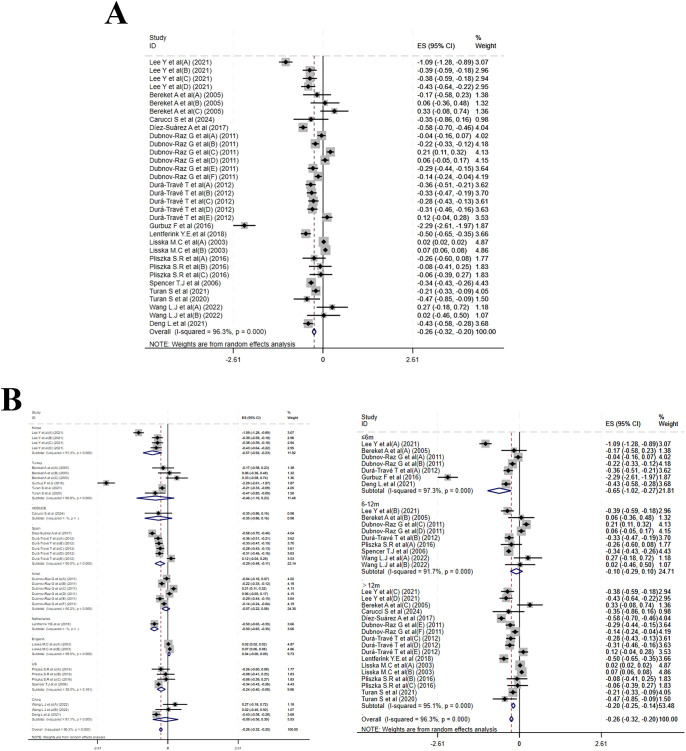
Meta-analysis results of BMI z-scores. **(A)** Forest plot of BMI z-scores; **(B)** Subgroup analysis of BMI z-scores by country and follow-up time.

Fifteen studies reported changes in BMI Z-score. Compared to baseline, MPH treatment led to a marked reduction in BMI Z-score (MD = –0.26, 95% CI: –0.32 to –0.20; I² = 96.3%, p < 0.001)(**Figure 6A**). Subgroup analysis by treatment duration displayed MDs of –0.65 (–1.02 to –0.27; I² = 97.3%, p < 0.001) for ≤ 6 months, –0.10 (–0.29 to 0.10; I² = 91.7%, p < 0.001) for 6–12 months, and –0.20 (–0.25 to –0.14; I² = 95.1%, p < 0.001) for >12 months. Country-stratified results indicated the following estimates: a pooled MD of –0.24 (–0.40 to –0.09; I² = 38.5%, p = 0.181) from two studies in the USA; –0.46 (–1.16 to 0.23; I² = 96.8%, p < 0.001) from four studies in Turkey; –0.09 (–0.56 to 0.39; I² = 81.1%, p = 0.006) from two studies in China; –0.29 (–0.48 to –0.11; I² = 90.0%, p < 0.001) from two studies in Spain; –0.57 (–0.92 to –0.23) from one study in Korea; –0.07 (–0.22 to 0.08) from one study in Israel; 0.04 (–0.00 to 0.09) from one study in United Kingdom; –0.05 (–0.65 to –0.35) from one study in the Netherlands; and –0.35 (–0.86 to 0.16) from one multicenter study (**Figure 6B**). Meta-regression of baseline age, sample size, and publication year identified no significant source of heterogeneity (**Supplementary Material 6.3**).

**Figure 7:**
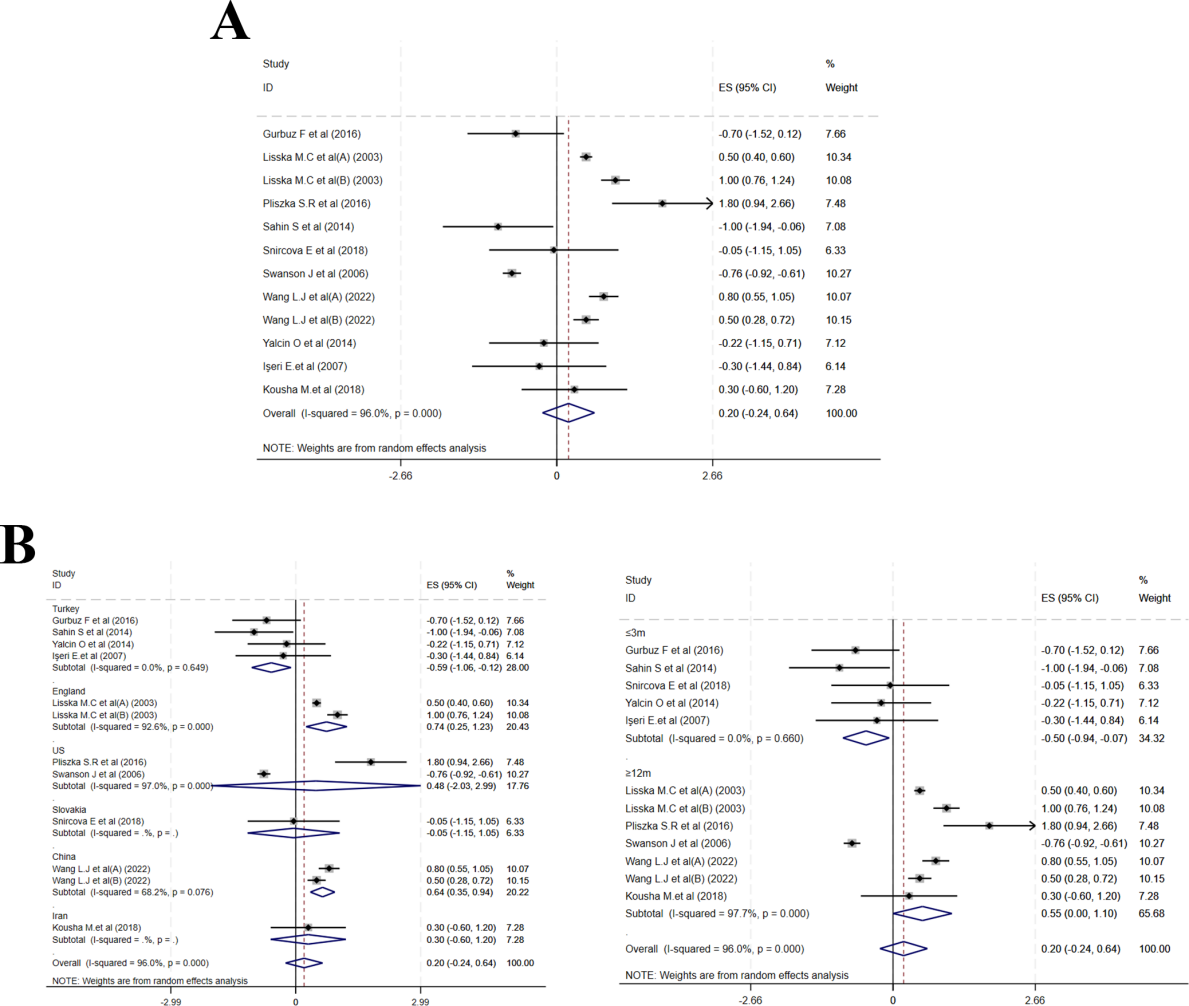
Meta-analysis results of mean BMI. **(A)** Forest plot of mean BMI; **(B)** Subgroup analysis of mean BMI by country and follow-up time.

Ten studies reported changes in mean BMI. The pooled effect did not reach statistical significance (MD = 0.20, 95% CI: –0.24 to 0.64; I² = 96.0%, p = 0.449) (**Figure 7A**). Subgroup analysis by treatment duration yielded MDs of –0.50 (–0.94 to –0.07; I² = 0.0%, P = 0.660) for ≤ 3 months and 0.55 (0.00 to 1.10; I² = 97.7%, p < 0.001) for > 12 months. Country-based subgroup results were as follows: a pooled MD of –0.59 (–1.06 to –0.12; I² = 0.0%, p = 0.649) from four studies in Turkey; 0.48 (–2.03 to 2.99; I² = 97.0%, p < 0.001) from two studies in the USA; 0.74 (0.25 to 1.23) from one study in United Kingdom; 0.64 (0.35 to 0.94) from one study in China; 0.30 (–0.60 to 1.20) from one study in Iran; and –0.05 (–1.15 to 1.05) from one study in Slovakia (**Figure 7B**). Meta-regression of baseline age, sample size, and publication year identified no significant source of heterogeneity (**Supplementary Material 6.4**).

#### 3.4.4 Sex subgroups

**Figure 8:**
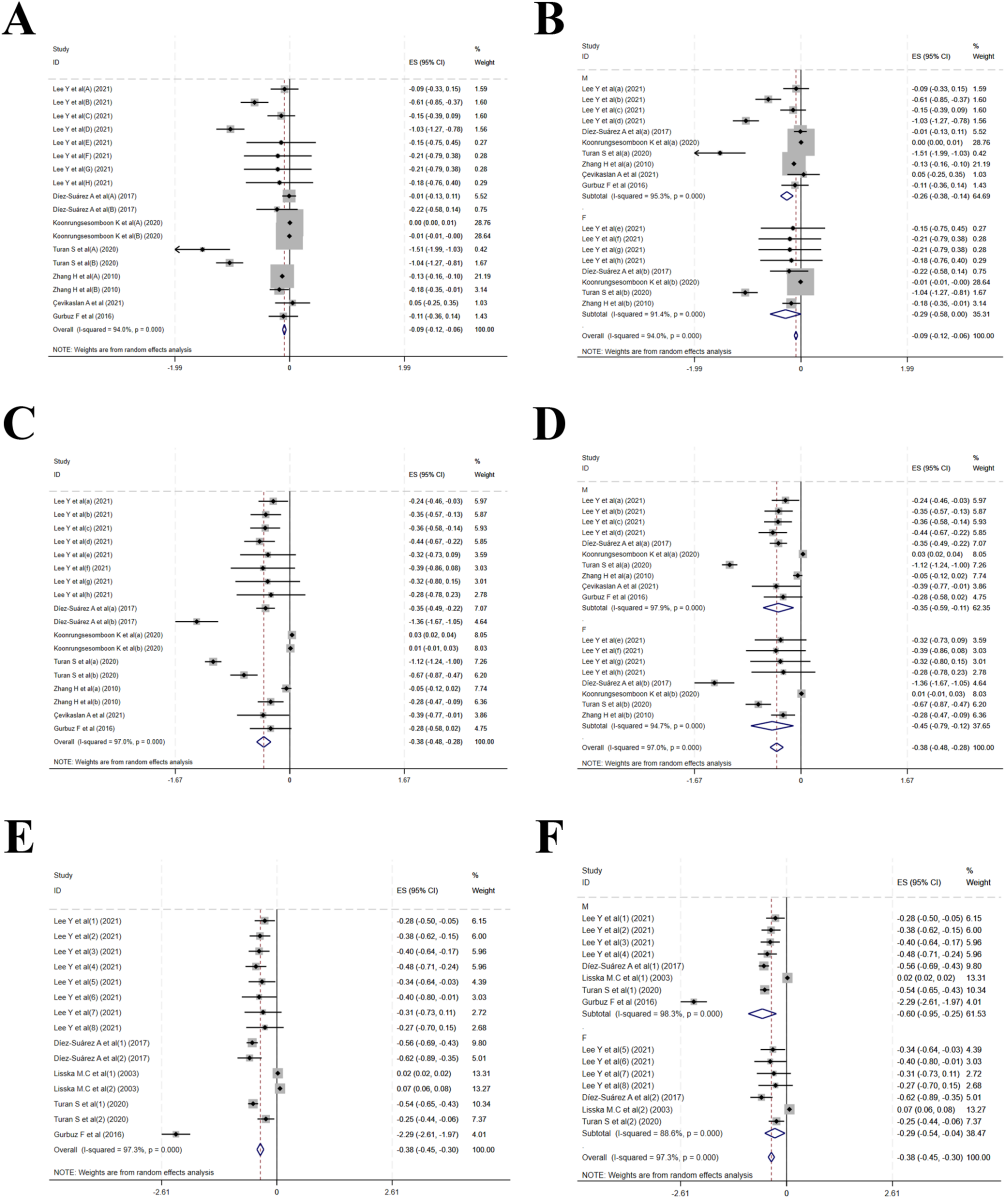
Sex-stratified analysis. **(A)** Forest plot of height z-scores; **(B)** Subgroup analysis of height z-scores by sex; **(C)** Forest plot of weight z-scores; **(D)** Subgroup analysis of weight z-scores by sex; **(E)** Forest plot of BMI z-scores; **(F)** Subgroup analysis of BMI z-scores by sex.

Five studies reporting sex-stratified changes in HZS and WZS before and after MPH treatment, alongside two studies that exclusively enrolled male children, were encompassed in the analysis. A marked reduction in HZS was observed post-treatment (MD = -0.09, 95% CI: -0.12 to -0.06; I² = 94.0%, p < 0.001)(**Figure 8A**). Subgroup analysis yielded MDs of -0.26 (-0.38 to -0.14; I² = 95.3%, P < 0.001) for males and -0.29 (-0.58 to 0.00; I² = 91.4%, P < 0.001) for females(**Figure 8B**). WZS was also decreased notably (MD = -0.38, 95% CI: -0.48 to -0.28; I² = 97.0%, p < 0.001)(**Figure 8C**). Subgroup results displayed MDs of -0.35 (-0.59 to -0.11; I² = 97.9%, P < 0.001) for males and -0.45 (-0.79 to -0.12; I² = 94.7%, P < 0.001) for females (**Figure 8D**).

Four studies reporting changes in sex-stratified BMI Z-score and one study that exclusively enrolled males were encompassed in the analysis. A notable decrease in BMI Z-score was found (MD = -0.38, 95% CI: -0.45 to -0.30; I² = 94.0%, p < 0.001)(**Figure 8E**). Subgroup results displayed MDs of -0.60 (-0.95 to -0.25; I² = 98.3%, P < 0.001) for males and -0.29 (-0.54 to -0.04; I² = 88.6%, P < 0.001) for females. Sex was not identified as a source of heterogeneity (**Figure 8F**).

### 3.5 Sensitivity analysis

**Figure 9:**
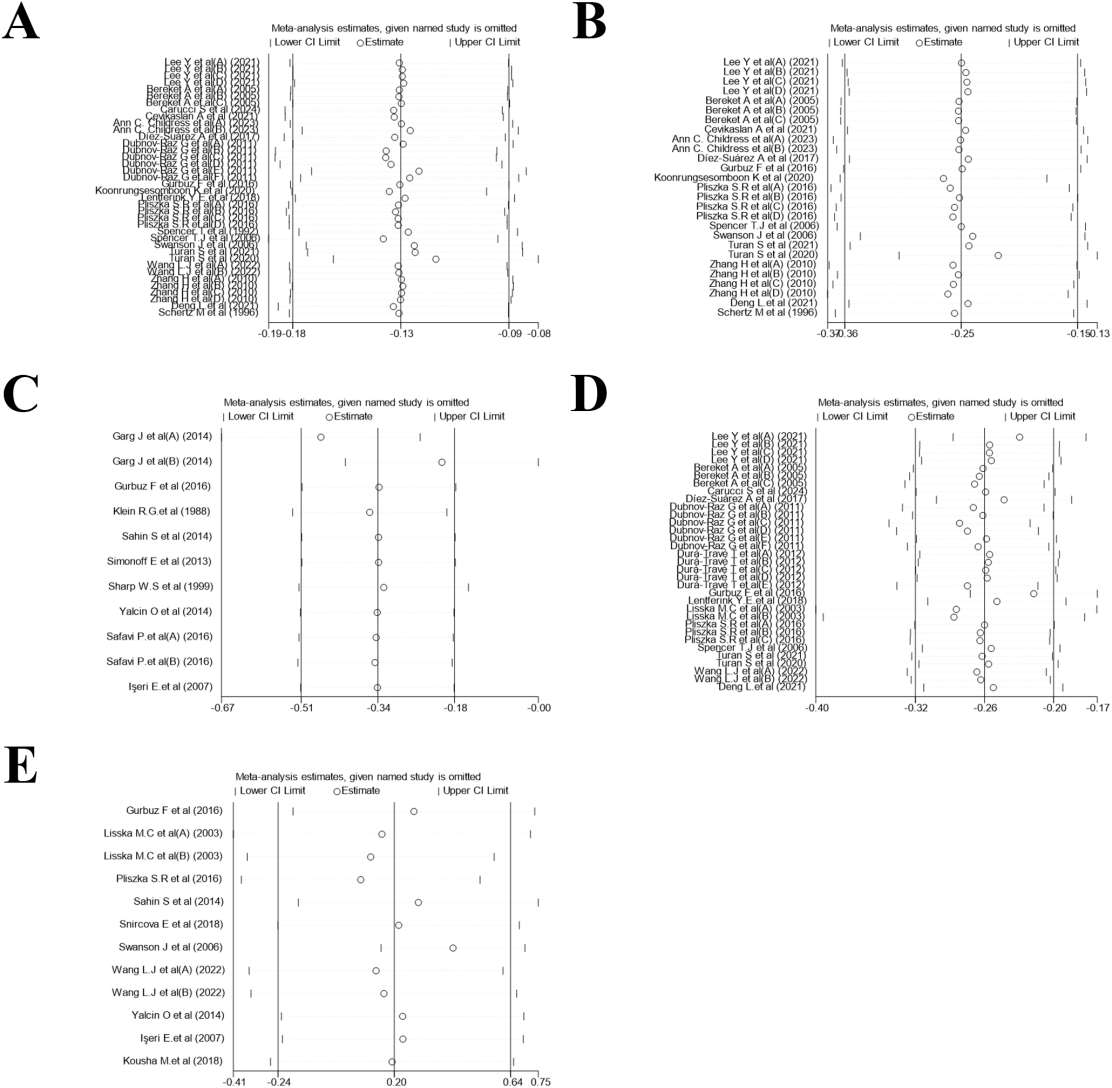
Sensitivity analysis. **(A)** Height z-scores; **(B)** Weight z-scores; **(C)** Mean weight; **(D)** BMI z-scores; **(E)** Mean BMI.

Leave-one-out sensitivity analyses for each outcome demonstrated that no individual investigation demonstrated a significant impact on the combined effect estimate, indicating good result stability (**Figure 9**).

### 3.6 Publication bias

**Figure 10:**
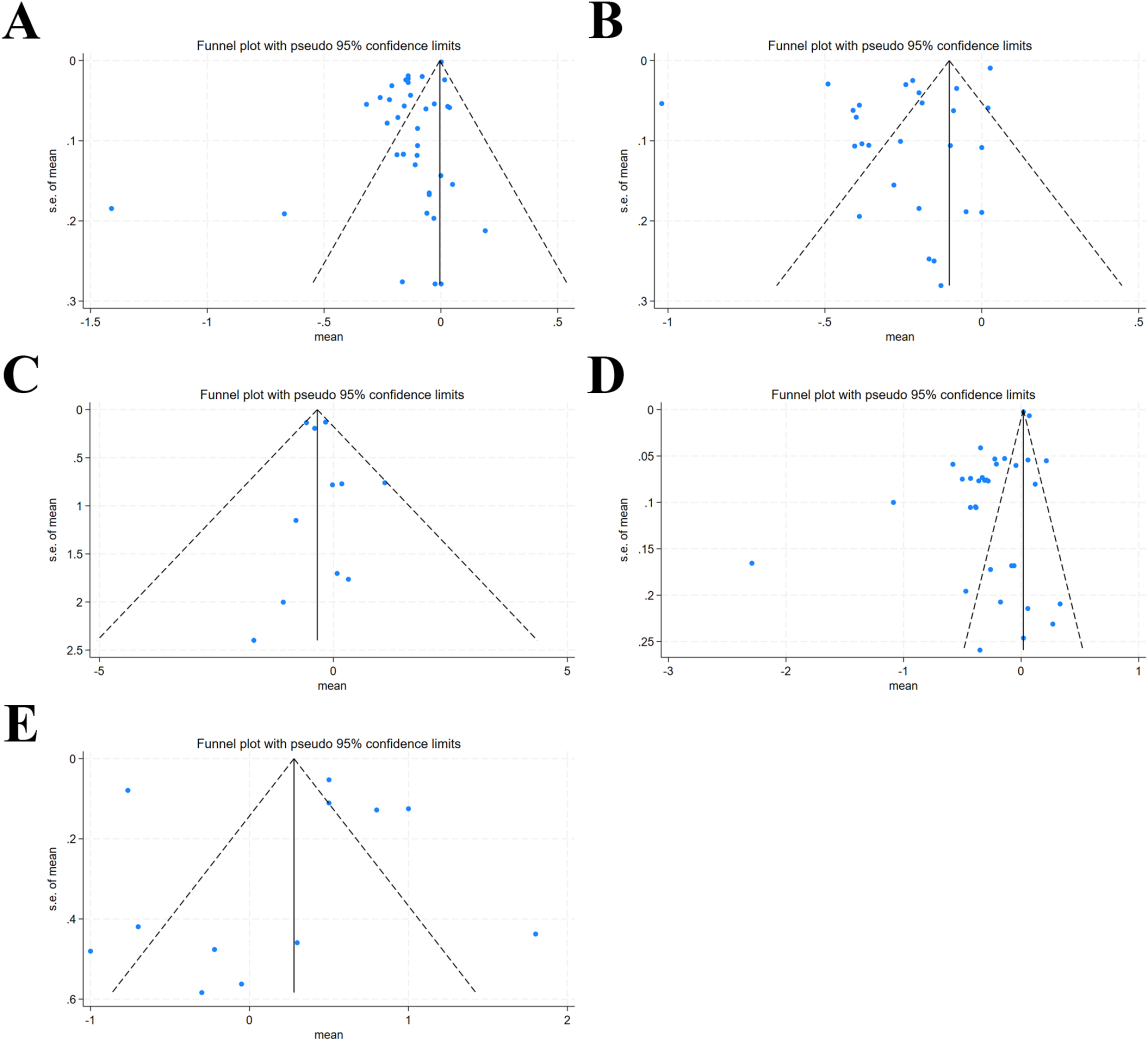
Funnel plot for publication bias. **(A)** Height z-scores; **(B)** Weight z-scores; **(C)** Mean weight; **(D)** BMI z-scores; **(E)** Mean BMI.

Egger’s test and Funnel plots were applied to assess publication bias for all outcomes. Significant publication bias was detected for HZS (p < 0.001), WZS (p = 0.012), and BMI Z-score (p < 0.001), consistent with Egger’s test (P < 0.05) (**Figure 10**; **Supplementary Material 7**). The trim-and-fill approach was adopted for adjustment; results did not reverse post-adjustment, suggesting that the detected bias did not undermine result reliability (**Supplementary Material 8**).

## 4 Discussion

Our meta-analysis systematically appraised the effect of MPH monotherapy on physical growth parameters in children and adolescents with ADHD. The findings indicate that MPH treatment may be linked to growth deficits because marked differences in weight, BMI, and height were noted before and after treatment. The most pronounced change was observed in weight. Nevertheless, no definitive influencing factors were identified. Although extended pharmacotherapy is often theoretically required for patients, the potential for growth deficits warrants clinical vigilance.

For height, MPH treatment led to a notable reduction in HZS compared to baseline, aligning with prior meta-analytic results (56). Numerous clinical studies associate MPH with potential growth deficits. However, in terms of height, these reductions are considered to be of small magnitude. This suggests a clinical presentation more consistent with mild deceleration of growth rather than pronounced impairment of height, an effect that may attenuate over time. This perspective is supported by a two-year prospective Attention Deficit Disorder Drug Use Chronic Effects study conducted by the European Commission (57). MPH is a central stimulant. Its dopaminergic and adrenergic effects may suppress the release of insulin-like growth factor (25). Moreover, MPH modulates central dopamine concentration, potentially interfering with the secretion of growth hormones—a key regulator of linear growth in children. This interference may explain the possibility of catch-up growth following the discontinuation of stimulants (35). The long-term influence of MPH on height and its underlying mechanisms require further investigation.

A recent large-scale cohort study with follow-up through adulthood (58) finds that although the reduction in adult height associated with MPH treatment is significant, the absolute difference is less than 1 cm (PES < 0.001), indicating limited clinical relevance. Notably, despite a modest overall effect size, this mild deceleration of growth may remain clinically relevant in certain individuals with lower baseline height or those undergoing rapid pubertal growth. Given that MPH is frequently prescribed for extended periods in clinical practice, the present research included participants across a wide age range, with follow-up extending up to five years. This suggests that a considerable proportion of patients experience medication exposure covering the entire pubertal period. Thus, expecting catch-up growth after the cessation of treatment as a universal outcome appears unrealistic, thereby posing a potential risk to final adult height among long-term users. Thus, the possible influence on specific high-risk subgroups should not be overlooked solely based on a small overall effect.

Regarding weight, WZS decreased notably post-treatment, accompanied by short-term weight loss. This is consistent with previous research (59). MPH may induce direct, short-term weight loss in pediatric patients, as directly reported in most studies. However, over the long term, the pooled effect size for the decrease in WZS (MD = −0.25) is statistically significant but of modest overall clinical magnitude. The suppressive effect of MPH on weight may also exhibit a degree of time dependence and reversibility. A retrospective observational study from Thailand reports that WZS decreases from 0.1262 to 0.1177 in the first year of treatment, recovers in the second year, and continues increasing in the third and fourth years (36). This pattern may be related to the lower doses of MPH employed. Overall, current evidence suggests that the impact of MPH on weight more closely resembles an early, pronounced deviation of growth that diminishes over time, rather than sustained, progressive long-term suppression of weight.

Appetite suppression, a common adverse effect of MPH, leads to reduced food intake. Chronic deficiencies in energy, protein, calcium, and other crucial nutrients likely contribute directly to weight loss. Conversely, limited evidence links nutritional deficiencies to growth retardation during MPH treatment (60), potentially reflecting individual variability in response to stimulants (61). Furthermore, the inherent heterogeneity of ADHD symptoms in children can lead to pre-existing dietary behavior issues. These issues may interact with side effects of medication, making it difficult to distinguish their respective causal contributions. The available evidence is insufficient to suggest that most children will experience clinically severe malnutrition or require widespread treatment discontinuation solely due to mild weight loss. The short-term adverse effects of MPH on weight appear to be offset by its anticipated benefits, and no specific guidelines recommend modifying treatment solely due to weight loss. The potential for more pronounced suppression of weight and appetite in a minority of individuals warrants genuine concern.

BMI Z-score decreased significantly following MPH treatment, corroborating most prior clinical findings. This does not, however, indicate a state of nutritional wasting. Moreover, the pooled effect for mean BMI change is not statistically significant. For BMI, the clinical significance of a small effect size is highly individualized and largely depends on the patient’s baseline physical status. One study compares BMI changes in normal-weight versus overweight/obese children after the initiation of MPH treatment. Following one to three years of treatment, the reduction in BMI-SDS is significantly greater in the overweight/obese group compared with normal-weight peers (62). However, not all studies have observed a decrease in BMI Z-score (51). Some investigations have explored the relationship between ADHD and overweight/obesity in children and adolescents. Anderson et al. (63) report no statistically significant difference in BMI Z-score between children with ADHD and their normal peers. Nonetheless, most studies suggest that children with ADHD may have a higher obesity rate, but they do not necessarily exhibit elevated overweight rates or mean BMI (64, 65). This discrepancy may stem from variations in the expression of ADHD symptoms. Impulsivity and inattention can lead to dysregulated eating patterns, increasing risks for overweight and obesity (66). Additionally, ADHD-related academic difficulties and stress negatively affect dietary habits and sleep, subsequently impacting nutritional status (67).

In clinical practice, BMI is frequently used as an indicator of individual nutritional status. For patients with a higher baseline BMI, a reduction in BMI Z-score may reflect a shift toward a healthier range of BMI, potentially representing a neutral or even beneficial effect (68). Conversely, for children with a baseline BMI already in a lower percentile, such a reduction may place them at risk of underweight, raising actual clinical concern.

Although none of the studies included in this research reported bone mineral density (BMD) as an outcome measure, skeletal health remains equally important for medication-related growth safety. Carucci et al. (26) supplement this perspective by evaluating bone maturation using assessments of bone age following long-term exposure to MPH. They find only mild acceleration of bone maturation after 24 months of treatment. Existing literature presents inconsistent findings regarding changes in BMD. Fu et al. (69) and Poulton et al. (70) report an association between exposure to stimulants and slower bone mass accrual or reduced BMD, whereas Lahat et al. (71) observe no significant difference in BMD following treatment with MPH. These discrepancies may stem from methodological differences and population characteristics. A recent review encompassing five non-randomized studies (72) concludes that childhood exposure to psychostimulants correlates with impaired BMD. Nevertheless, given that most available evidence is not strictly limited to MPH monotherapy, extrapolation of these results requires caution. Mechanistically, MPH may influence the accumulation of bone mass and metabolism of the bone through reduced intake of nutrients or indirect sympathomimetic effects. BMI, a marker of nutritional status, is closely correlated with bone mass accrual. In view of the declines in weight and BMI z-scores noted in the present research, we propose that current evidence suggests MPH-related growth effects extend beyond linear growth, and the potential impact of such an effect on skeletal health should be further investigated.

Based on the foregoing discussion, future research should adopt more explicit and standardized methodological strategies. Specifically, prospective longitudinal designs are recommended, with systematic reporting of various physical growth outcomes at standardized assessment time points, along with clear documentation of formulation, dosage, cumulative exposure, and treatment interruptions of MPH. Additionally, important individual-level factors that may influence growth trajectories—such as baseline nutritional status, pubertal stage, and bone age or BMD—should be fully incorporated to more comprehensively assess the long-term effects of MPH on growth and developmental safety. Future studies should also extend the durations of follow-up where possible, or conduct long-term longitudinal investigations that stratify participants by pubertal stage and assess final adult height, thereby better distinguishing transient deceleration of growth from persistent long-term effects.

## 5 Strengths and limitations

The present research focused solely on the influence of MPH monotherapy on growth parameters. Studies involving concurrent psychotropic medications or specified behavioral therapies were excluded from the analysis. This approach aimed to isolate the potential growth deficit effect of the drug itself and assess its persistence. Furthermore, this research diverged from prior analyses of single outcomes by encompassing changes in weight, BMI, and height, providing a multifaceted explanation for the influence of MPH on different growth indicators. The inclusion of study populations from Asia, Europe, and America reflects the influence of MPH across diverse ethnicities to some extent.

Several limitations should be acknowledged. First, the analysis predominantly encompassed observational studies. Although prospective and retrospective designs were equally represented, the proportion of RCTs was low. Therefore, the pooled effect estimates were inevitably subject to limitations inherent to non-randomized studies, including confounding, selection, and information biases. Variability in study populations with respect to symptomatology, comorbid conditions, and baseline nutritional status—all factors that may influence growth trajectories of children—could not be uniformly controlled across studies. Furthermore, treatment-related factors, such as dose adjustment of MPH and medication adherence, were inconsistently reported in the included studies. Substantial heterogeneity was present for most outcomes. While a random-effects model was applied for adjustment, no clear sources were identified. Beyond inadequate analysis of confounding effects from various variables, this may also reflect methodological heterogeneity across studies. Second, although subgroup analyses by dose and formulation were planned, methodological inconsistencies—such as unclear formulation distinctions, unreported dose adjustment processes, and non-uniform units—precluded such analyses. Third, existing studies lack long-term follow-up data. Definitive conclusions on the long-term influence of MPH on final adult height and weight cannot be drawn. Fourth, publication bias may have influenced the effect estimates in the present analysis. The evidence included is derived primarily from published literature, without systematic inclusion of grey literature or unpublished studies. Consequently, results that are statistically significant or suggestive of growth suppression are more likely to be published, possibly leading to overestimation of the pooled effect sizes. Although publication bias was assessed using funnel plots and Egger’s test, the latter revealed notable publication bias for most outcome measures. Despite adjustment via the trim-and-fill method, the inherent high heterogeneity among eligible studies precludes complete reconstruction of unpublished data. This potentially introduces some controversy into the final pooled effect estimates. Accordingly, the results should be interpreted with caution. Fifth, only English-language studies were included, which may have introduced language bias. This consideration is particularly important given that subgroup analyses stratified by country were conducted. The resulting differences, while possibly reflecting genuine regional variations, may also be partially influenced by language restrictions and the limited representativeness of the evidence. Thus, these findings should be interpreted with due caution. Finally, most studies included wide age ranges. This indicated that many participants were approaching or undergoing puberty—a period of marked growth spurts and substantial individual variation. Only three eligible studies (14, 37, 54) performed age stratification, and the cut-off points were inconsistent. Such inconsistency complicated the analysis stratified by specific pubertal status or age.

In conclusion, this meta-analysis demonstrates a notable correlation of MPH use with reduced growth parameters in children and adolescents with ADHD. Although the effect sizes are modest, they are not negligible, particularly for children requiring long-term pharmacotherapy. When initiating treatment, clinicians should prioritize individualized management. Monitoring growth curves and considering individual growth trajectories is advisable. For individuals showing clear deceleration in height or weight gain, ‘drug holidays’ may be considered to mitigate potential long-term adverse effects. Future research should prioritize prospective designs with standardized protocols to further elucidate the mechanisms underlying MPH-related growth effects and their impact on long-term developmental outcomes, particularly adult height. Assessing growth parameters before treatment initiation and conducting regular monitoring with curve plotting are feasible measures for ensuring a favorable risk-benefit balance of pharmacotherapy for individuals with ADHD.

## Data Availability

The original contributions presented in the study are included in the article/**Supplementary Material**. Further inquiries can be directed to the corresponding author.

## Supplementary material

Supplementary Material 1 PRISMA guideline.

Supplementary Material 2 Search strategy. 2.1 Database: PubMed <inception to December 16, 2025> 2.2 Database: Embase < inception to December 16, 2025> 2.3 Database: Cochrane < inception to December 16, 2025 > 2.4 Database: Web of Science < inception to December 16, 2025 >.

Supplementary Material 3 Basic characteristics of included studies.

Supplementary Material 4 The quality assessment of the cohort studies included in this study.

Supplementary Material 5 GRADE assessment result. 5.1 Height z-score 5.2 Weight z-score 5.3 Mean weight 5.4 Mean BMI.

Supplementary Material 6 Results of regression analysis. 6.1 Height z-score 6.2 Weight z-score 6.3 BMI z-score 6.4 Mean BMI.

Supplementary Material 7 Publication bias. 7.1 Height z-score 7.2 Weight z-score 7.3 BMI z-score.

Supplementary Material 8 Trim and filling method. 8.1 Height z-score 8.2 Weight z-score 8.3 BMI z-score.
