# Supplementary material for: Effect of methylphenidate on physical growth indicators in children and adolescents with attention-deficit/hyperactivity disorder: a systematic review and meta-analysis
Source: Front Psychiatry. 2026 May 11;17:1794403. doi: 10.3389/fpsyt.2026.1794403 (PMC13199321; doi:10.3389/fpsyt.2026.1794403)

Supplementary materials

**Contents:**

[Supplementary material 1: PRISMA guideline 2](#_Toc225437636)

[Supplementary material 2: Search Strategy 6](#_Toc225437637)

[2.1 Database: PubMed <inception to December 16, 2025> 6](#_Toc225437638)

[2.2 Database: Embase < inception to December 16, 2025> 7](#_Toc225437639)

[2.3 Database: Cochrane < inception to December 16, 2025 > 8](#_Toc225437640)

[2.4 Database: Web Of Science < inception to December 16, 2025 > 9](#_Toc225437641)

[Supplementary material 3: Basic characteristics of included studies 10](#_Toc225437642)

[Supplementary material 4: The quality assessment of cohort study included in this study 14](#_Toc225437643)

[Supplementary material 5: GRADE assessment result 16](#_Toc225437644)

[5.1 Height z score 16](#_Toc225437645)

[5.2 Weight z score 16](#_Toc225437646)

[5.3 Mean weight 17](#_Toc225437647)

[5.4 Mean BMI 17](#_Toc225437648)

[Supplementary material 6: Results of regression analysis 18](#_Toc225437649)

[6.1 Height z score 18](#_Toc225437650)

[6.2 Weight z score 18](#_Toc225437651)

[6.3 BMI z score 18](#_Toc225437652)

[6.4 Mean BMI 18](#_Toc225437653)

[Supplementary material 7: publication bias 19](#_Toc225437654)

[7.1 Height z score 19](#_Toc225437655)

[7.2 Weight z score 20](#_Toc225437656)

[7.3 BMI z score 21](#_Toc225437657)

[Supplementary material 8: Trim and filling method 22](#_Toc225437658)

[8.1 Height z score 22](#_Toc225437659)

[8.2 Weight z score 22](#_Toc225437660)

[8.3 BMI z score 23](#_Toc225437661)

# Supplementary material 1: PRISMA guideline

| **Section and Topic** | **Item #** | **Checklist item** | **Location where item is reported** |
| --- | --- | --- | --- |
| **TITLE** | | |  |
| Title | 1 | Identify the report as a systematic review. | Page 1 |
| **ABSTRACT** | | |  |
| Abstract | 2 | See the PRISMA 2020 for Abstracts checklist. | Page 1 |
| **INTRODUCTION** | | |  |
| Rationale | 3 | Describe the rationale for the review in the context of existing knowledge. | Page 2 |
| Objectives | 4 | Provide an explicit statement of the objective(s) or question(s) the review addresses. | Page 2 |
| **METHODS** | | |  |
| Eligibility criteria | 5 | Specify the inclusion and exclusion criteria for the review and how studies were grouped for the syntheses. | Page 2 |
| Information sources | 6 | Specify all databases, registers, websites, organisations, reference lists and other sources searched or consulted to identify studies. Specify the date when each source was last searched or consulted. | Page 2-3 |
| Search strategy | 7 | Present the full search strategies for all databases, registers and websites, including any filters and limits used. | Page 2-3,  Supplementary material 2 |
| Selection process | 8 | Specify the methods used to decide whether a study met the inclusion criteria of the review, including how many reviewers screened each record and each report retrieved, whether they worked independently, and if applicable, details of automation tools used in the process. | Page 3 |
| Data collection process | 9 | Specify the methods used to collect data from reports, including how many reviewers collected data from each report, whether they worked independently, any processes for obtaining or confirming data from study investigators, and if applicable, details of automation tools used in the process. | Page 3 |
| Data items | 10a | List and define all outcomes for which data were sought. Specify whether all results that were compatible with each outcome domain in each study were sought (e.g. for all measures, time points, analyses), and if not, the methods used to decide which results to collect. | Page 3 |
|  | 10b | List and define all other variables for which data were sought (e.g. participant and intervention characteristics, funding sources). Describe any assumptions made about any missing or unclear information. | Page 3 |
| Study risk of bias assessment | 11 | Specify the methods used to assess risk of bias in the included studies, including details of the tool(s) used, how many reviewers assessed each study and whether they worked independently, and if applicable, details of automation tools used in the process. | Page 3 |
| Effect measures | 12 | Specify for each outcome the effect measure(s) (e.g. risk ratio, mean difference) used in the synthesis or presentation of results. | Page 4 |
| Synthesis methods | 13a | Describe the processes used to decide which studies were eligible for each synthesis (e.g. tabulating the study intervention characteristics and comparing against the planned groups for each synthesis (item #5)). | Page 4 |
|  | 13b | Describe any methods required to prepare the data for presentation or synthesis, such as handling of missing summary statistics, or data conversions. | Page 4 |
|  | 13c | Describe any methods used to tabulate or visually display results of individual studies and syntheses. | Page 4 |
|  | 13d | Describe any methods used to synthesize results and provide a rationale for the choice(s). If meta-analysis was performed, describe the model(s), method(s) to identify the presence and extent of statistical heterogeneity, and software package(s) used. | Page 4 |
|  | 13e | Describe any methods used to explore possible causes of heterogeneity among study results (e.g. subgroup analysis, meta-regression). | Page 4 |
|  | 13f | Describe any sensitivity analyses conducted to assess robustness of the synthesized results. | Page 4 |
| Reporting bias assessment | 14 | Describe any methods used to assess risk of bias due to missing results in a synthesis (arising from reporting biases). | Page 4 |
| Certainty assessment | 15 | Describe any methods used to assess certainty (or confidence) in the body of evidence for an outcome. | Page 3-4 |
| **RESULTS** | | |  |
| Study selection | 16a | Describe the results of the search and selection process, from the number of records identified in the search to the number of studies included in the review, ideally using a flow diagram. | Figure 1 |
|  | 16b | Cite studies that might appear to meet the inclusion criteria, but which were excluded, and explain why they were excluded. | Page 4 |
| Study characteristics | 17 | Cite each included study and present its characteristics. | Page 4,  Supplementary material 3 |
| Risk of bias in studies | 18 | Present assessments of risk of bias for each included study. | Figure 2,  Supplementary material 4 |
| Results of individual studies | 19 | For all outcomes, present, for each study: (a) summary statistics for each group (where appropriate) and (b) an effect estimate and its precision (e.g. confidence/credible interval), ideally using structured tables or plots. | Figure 3-8 |
| Results of syntheses | 20a | For each synthesis, briefly summarise the characteristics and risk of bias among contributing studies. | Page 5-6 |
|  | 20b | Present results of all statistical syntheses conducted. If meta-analysis was done, present for each the summary estimate and its precision (e.g. confidence/credible interval) and measures of statistical heterogeneity. If comparing groups, describe the direction of the effect. | Page 5-7 |
|  | 20c | Present results of all investigations of possible causes of heterogeneity among study results. | Page 5-7 |
|  | 20d | Present results of all sensitivity analyses conducted to assess the robustness of the synthesized results. | Page 7, Figure 9 |
| Reporting biases | 21 | Present assessments of risk of bias due to missing results (arising from reporting biases) for each synthesis assessed. | Page 7, Figure 10, Supplementary material 7-8 |
| Certainty of evidence | 22 | Present assessments of certainty (or confidence) in the body of evidence for each outcome assessed. | Page 5, Supplementary material 5 |
| **DISCUSSION** | | |  |
| Discussion | 23a | Provide a general interpretation of the results in the context of other evidence. | Page 7-11 |
|  | 23b | Discuss any limitations of the evidence included in the review. | Page 11-12 |
|  | 23c | Discuss any limitations of the review processes used. | Page 11-12 |
|  | 23d | Discuss implications of the results for practice, policy, and future research. | Page 11-12 |
| **OTHER INFORMATION** | | |  |
| Registration and protocol | 24a | Provide registration information for the review, including register name and registration number, or state that the review was not registered. | Page 2 |
|  | 24b | Indicate where the review protocol can be accessed, or state that a protocol was not prepared. | Page 2 |
|  | 24c | Describe and explain any amendments to information provided at registration or in the protocol. | Page 2 |
| Support | 25 | Describe sources of financial or non-financial support for the review, and the role of the funders or sponsors in the review. | Page 13 |
| Competing interests | 26 | Declare any competing interests of review authors. | Page 13 |
| Availability of data, code and other materials | 27 | Report which of the following are publicly available and where they can be found: template data collection forms; data extracted from included studies; data used for all analyses; analytic code; any other materials used in the review. | Page 13 |

*From:*  Page MJ, McKenzie JE, Bossuyt PM, Boutron I, Hoffmann TC, Mulrow CD, et al. The PRISMA 2020 statement: an updated guideline for reporting systematic reviews. BMJ 2021;372:n71. doi: 10.1136/bmj.n71. This work is licensed under CC BY 4.0. To view a copy of this license, visit <https://creativecommons.org/licenses/by/4.0/>

# Supplementary material 2: Search Strategy

## 2.1 Database: PubMed <inception to December 16, 2025>

| #1 | "Attention Deficit Disorder with Hyperactivity"[Mesh] | 38,452 |
| --- | --- | --- |
| #2 | ("Attention Deficit Disorder with Hyperactivity"[Mesh]) OR ("ADDH"[Title/Abstract] OR "ADHD"[Title/Abstract] OR "attention deficit"[Title/Abstract] OR "Hyperkinetic Syndrome"[Title/Abstract] OR "Minimal Brain Dysfunction"[Title/Abstract]) | 57993 |
| #3 | "Methylphenidate"[Mesh] | 8183 |
| #4 | ("Methylphenidate"[Mesh]) OR ("Tsentedrin"[Title/Abstract] OR "Centedrin"[Title/Abstract] OR "Phenidylate"[Title/Abstract] OR "Ritalin*"[Title/Abstract] OR "Metadate"[Title/Abstract] OR "Equasym"[Title/Abstract] OR "Daytrana"[Title/Abstract] OR "Concerta"[Title/Abstract] OR "Addepta"[Title/Abstract] OR "Adhansia"[Title/Abstract] OR "Affenid"[Title/Abstract] OR "Aptensio"[Title/Abstract] OR "Atenza"[Title/Abstract] OR "Attenta"[Title/Abstract] OR "Benjorna"[Title/Abstract] OR "Biphentin"[Title/Abstract] OR "Cotempla"[Title/Abstract] OR "Delmosart"[Title/Abstract] OR "Difumenil"[Title/Abstract] OR "exattent xl"[Title/Abstract] OR "focusim xl"[Title/Abstract] OR "Foquest"[Title/Abstract] OR "Kinecteen"[Title/Abstract] OR "Kixel"[Title/Abstract] OR "matoride xl"[Title/Abstract] OR "Medanef"[Title/Abstract] OR "Medicebran"[Title/Abstract] OR "Medikinet"[Title/Abstract] OR "Mefinad"[Title/Abstract] OR "Meflynate"[Title/Abstract] OR "Methy*"[Title/Abstract] OR "Metyrol"[Title/Abstract] OR "Motiron"[Title/Abstract] OR "penidphenidyl hydrochloride"[Title/Abstract] OR "Quasym"[Title/Abstract] OR "Quillichew"[Title/Abstract] OR "Quillivant"[Title/Abstract] OR "Relexxii"[Title/Abstract] OR "Rilatine"[Title/Abstract] OR "Ritaphen"[Title/Abstract] OR "Rubicrono"[Title/Abstract] OR "Rubifen"[Title/Abstract] OR "rubifen retard"[Title/Abstract] OR "Tranquilyn"[Title/Abstract] OR "Tuzulby"[Title/Abstract] OR "xaggitin xl"[Title/Abstract] OR "xenidate xl"[Title/Abstract]) | 772450 |
| #5 | "Body Mass Index"[Mesh] | 164474 |
| #6 | ("Body Mass Index"[Mesh]) OR ("BMI"[Title/Abstract] OR "body ban mass"[Title/Abstract] OR "body mass"[Title/Abstract] OR "Quetelet* Index"[Title/Abstract] OR "Dwarfism"[Title/Abstract] OR "Nanism"[Title/Abstract] OR "weight"[Title/Abstract] OR "height"[Title/Abstract] OR "stature"[Title/Abstract] OR "growth"[Title/Abstract] OR "body length"[Title/Abstract]) | 3366745 |
| #7 | #2 and #4 and #6 | 499 |

## 2.2 Database: Embase < inception to December 16, 2025>

| #1 | 'attention deficit hyperactivity disorder'/exp | 94980 |
| --- | --- | --- |
| #2 | 'addh':ti,ab,kw OR 'adhd':ti,ab,kw OR 'attention deficit':ti,ab,kw OR 'hyperkinetic syndrome':ti,ab,kw OR 'minimal brain dysfunction':ti,ab,kw | 74806 |
| #3 | #1 OR #2 | 103723 |
| #4 | 'methylphenidate'/exp | 27522 |
| #5 | 'tsentedrin':ti,ab,kw OR 'centedrin':ti,ab,kw OR 'phenidylate':ti,ab,kw OR 'ritalin*':ti,ab,kw OR 'metadate':ti,ab,kw OR 'equasym':ti,ab,kw OR 'daytrana':ti,ab,kw OR 'concerta':ti,ab,kw OR 'addepta':ti,ab,kw OR 'adhansia':ti,ab,kw OR 'affenid':ti,ab,kw OR 'aptensio':ti,ab,kw OR 'atenza':ti,ab,kw OR 'attenta':ti,ab,kw OR 'benjorna':ti,ab,kw OR 'biphentin':ti,ab,kw OR 'cotempla':ti,ab,kw OR 'delmosart':ti,ab,kw OR 'difumenil':ti,ab,kw OR 'exattent xl':ti,ab,kw OR 'focusim xl':ti,ab,kw OR 'foquest':ti,ab,kw OR 'kinecteen':ti,ab,kw OR 'kixel':ti,ab,kw OR 'matoride xl':ti,ab,kw OR 'medanef':ti,ab,kw OR 'medicebran':ti,ab,kw OR 'medikinet':ti,ab,kw OR 'mefinad':ti,ab,kw OR 'meflynate':ti,ab,kw OR 'methy*':ti,ab,kw OR 'metyrol':ti,ab,kw OR 'motiron':ti,ab,kw OR 'penidphenidyl hydrochloride':ti,ab,kw OR 'quasym':ti,ab,kw OR 'quillichew':ti,ab,kw OR 'quillivant':ti,ab,kw OR 'relexxii':ti,ab,kw OR 'rilatine':ti,ab,kw OR 'ritaphen':ti,ab,kw OR 'rubicrono':ti,ab,kw OR 'rubifen':ti,ab,kw OR 'rubifen retard':ti,ab,kw OR 'tranquilyn':ti,ab,kw OR 'tuzulby':ti,ab,kw OR 'xaggitin xl':ti,ab,kw OR 'xenidate xl':ti,ab,kw | 935889 |
| #6 | #4 OR #5 | 950475 |
| #7 | 'body mass'/exp | 801966 |
| #8 | 'bmi':ti,ab,kw OR 'body ban mass':ti,ab,kw OR 'body mass':ti,ab,kw OR 'quetelet* index':ti,ab,kw OR 'dwarfism':ti,ab,kw OR 'nanism':ti,ab,kw OR 'weight':ti,ab,kw OR 'height':ti,ab,kw OR 'stature':ti,ab,kw OR 'growth':ti,ab,kw OR 'body length':ti,ab,kw | 4396950 |
| #9 | #7 OR #8 | 4624820 |
| #10 | #3 AND #6 AND #9 | 1241 |

## 2.3 Database: Cochrane < inception to December 16, 2025 >

| #1 | MeSH descriptor: [Attention Deficit Disorder with Hyperactivity] explode all trees | 3986 |
| --- | --- | --- |
| #2 | ('ADDH' OR 'ADHD' OR 'attention deficit' OR 'Hyperkinetic Syndrome' OR 'Minimal Brain Dysfunction'):ti,ab,kw | 9477 |
| #3 | #1 or #2 | 9477 |
| #4 | MeSH descriptor: [Methylphenidate] explode all trees | 2009 |
| #5 | ('Tsentedrin' OR 'Centedrin' OR 'Phenidylate' OR 'Ritalin*' OR 'Metadate' OR 'Equasym' OR 'Daytrana' OR 'Concerta' OR 'Addepta' OR 'Adhansia' OR 'Affenid' OR 'Aptensio' OR 'Atenza' OR 'Attenta' OR 'Benjorna' OR 'Biphentin' OR 'Cotempla' OR 'Delmosart' OR 'Difumenil' OR 'exattent xl' OR 'focusim xl' OR 'Foquest' OR 'Kinecteen' OR 'Kixel' OR 'matoride xl' OR 'Medanef' OR 'Medicebran' OR 'Medikinet' OR 'Mefinad' OR 'Meflynate' OR 'Methy*' OR 'Metyrol' OR 'Motiron' OR 'penidphenidyl hydrochloride' OR 'Quasym' OR 'Quillichew' OR 'Quillivant' OR 'Relexxii' OR 'Rilatine' OR 'Ritaphen' OR 'Rubicrono' OR 'Rubifen' OR 'rubifen retard' OR 'Tranquilyn' OR 'Tuzulby' OR 'xaggitin xl' OR 'xenidate xl'):ti,ab,kw | 29382 |
| #6 | #4 OR #5 | 29397 |
| #7 | MeSH descriptor: [Body Mass Index] explode all trees | 14211 |
| #8 | ('BMI' OR 'body ban mass' OR 'body mass' OR 'Quetelet* Index' OR 'Dwarfism' OR 'Nanism' OR 'weight' OR 'height' OR 'stature' OR 'growth' OR 'body length'):ti,ab,kw | 293660 |
| #9 | #7 OR #8 | 293660 |
| #10 | #3 AND #6 AND #9 | 266 |

## 2.4 Database: Web Of Science < inception to December 16, 2025 >

| #1 | TS=((ADDH) OR (ADHD) OR (attention deficit) OR (Hyperkinetic Syndrome) OR (Minimal Brain Dysfunction)) | 78795 |
| --- | --- | --- |
| #2 | TS=((Tsentedrin) OR (Centedrin) OR (Phenidylate) OR (Ritalin*) OR (Metadate) OR (Equasym) OR (Daytrana) OR (Concerta) OR (Addepta) OR (Adhansia) OR (Affenid) OR (Aptensio) OR (Atenza) OR (Attenta) OR (Benjorna) OR (Biphentin) OR (Cotempla) OR (Delmosart) OR (Difumenil) OR (exattent xl) OR (focusim xl) OR (Foquest) OR (Kinecteen) OR (Kixel) OR (matoride xl) OR (Medanef) OR (Medicebran) OR (Medikinet) OR (Mefinad) OR (Meflynate) OR (Methy*) OR (Metyrol) OR (Motiron) OR (penidphenidyl hydrochloride) OR (Quasym) OR (Quillichew) OR (Quillivant) OR (Relexxii) OR (Rilatine) OR (Ritaphen) OR (Rubicrono) OR (Rubifen) OR (rubifen retard) OR (Tranquilyn) OR (Tuzulby) OR (xaggitin xl) OR (xenidate xl)) | 1142386 |
| #3 | TS=((BMI) OR (body ban mass) OR (body mass) OR (Quetelet* Index) OR (Dwarfism) OR (Nanism) OR (weight) OR (height) OR (stature) OR (growth) OR (body length)) | 5507113 |
| #4 | #1 AND #2 AND #3 | 727 |

# Supplementary material 3: Basic characteristics of included studies

| **ID** | **Title** | **Author** | **Year** | **Country** | **Study design** | **MPH** | **N(M/F)** | **Age (year)** | **Control group** | **Other group** | **Diagnostic criteria for ADHD** | **Dosage** | **Course of treatment** | **Measurement time** | **Outcome indicator** | **NOS score** |
| --- | --- | --- | --- | --- | --- | --- | --- | --- | --- | --- | --- | --- | --- | --- | --- | --- |
| 1 | A 24-Month Effects of Methylphenidate Use on Growth in Children and Adolescents With Attention Deficit Hyperactivity Disorder | Lee Y et al | 2021 | Korea | Retrospective cohort study | osmotic controlled-release, extended-release, or immediate-release | 82(64/18) | 8.17±1.93 |  | <9 y(57), 9-12 y(19), ≥12 y(6) | DSM-IV-TR or DSM 5th |  | 24m | Every 6m | height, weight, BMI z-score | 8 |
| 2 | Height, weight, IGF-I, IGFBP-3 and thyroid functions in prepubertal children with attention deficit hyperactivity disorder: effect of methylphenidate treatment | Bereket A et al | 2005 | Turkey | Prospective Study |  | 14(10/4) | 8.12±1.8 (6.47-10.32) |  |  | DSM-IV | initial dose:0.75 mg/kg/d | 16m | Every 4m | height, weight, BMI z-score | 5 |
| 3 | The Impact of Methylphenidate on Pubertal Maturation and Bone Age in ADHD Children and Adolescents: Results from the ADHD Drugs Use Chronic Effects (ADDUCE) Project | Carucci S et al | 2024 | Multicenter: Germany, Hungary, Italy, the United Kingdom, the Netherlands | Prospective Study | immediate-release, 15 subjects switched to a sustained release patients | 756(622/134) Bone Age sample:25 | 8.85±1.86 |  |  | DSM-IV | initial dose:0.25 ± 0.07 mg/kg/d, maintenance dose:0.71 ± 0.32 mg/kg/d | 24m | Every 12m | height, BMI z-score | 9 |
| 4 | Effects of methylphenidate on height, weight and blood biochemistry parameters in prepubertal boys with attention deficit hyperactivity disorder: an open label prospective study | Çevikaslan A et al | 2021 | Turkey | Prospective Study | OROS-Methylphenidate(77.4%)or IR-Methylphenidate(22.5%) | 31 males | 87.6m±9.2 (6.2-8.7) |  | ADHD-C:18, ADHD-I:7, ADHD-H:6 | DSM-5 | OROS-MPH: 18 mg/ d,  IR-MPH: 5 mg, three times a d. Fixed dose.  Mean dose: 0.66±0.12 mg/kg/d | 6m | Twice(baseline and 6th month) | height, weight z-score | 5 |
| 5 | Analysis of Growth Velocity in Children with Attention-Deficit/Hyperactivity Disorder Treated for up to 12 m with Serdexmethylphenidate/Dexmethylphenidate | Childress A.C et al | 2023 | US 18 regions | Prospective Study | SDX/d-MPH (sustained release) | 238(145/93) | 9.1±1.87 |  |  | DSM-5 | 39.2/7.8 mg/d | 12m | Monthly | height, weight mean/z-score/percentile | 5 |
| 6 | Weight, Height, and Body Mass Index in Patients with Attention-Deficit/Hyperactivity Disorder Treated with Methylphenidate | Díez-Suárez A et al | 2017 | Spain | Retrospective cohort study | pellets, osmotic release and immediate release | 342(274/68) | 10.7±3.84 |  | Children: 6–12 y(249)(68.6%) Adolescents: 13–18 y(114)(31.4%) | DSM-IV-TR | Mean dose: 1.25±0.40 mg/(kg.d),59.6±22.9 mg/d | 27(14-41)m | Twice(baseline and follow-up) | height, weight, BMI z-score | 5 |
| 7 | Body mass index of children with attention-deficit/hyperactivity disorder | Dubnov-Raz G et al | 2011 | Israel(Hadassah) | Retrospective cohort study | Regular: n = 52 Slow-release/Long-acting: n = 61 Osmotic-release MPH: n = 22 | 275(200/75) | 10.4±2.4 | Healthy controls: 51 | First Methylphenidate Untreated (n=140) First Methylphenidate Treated (n=135) | DSM-IV-TR | Mean dose: 0.43±0.22mg/kg,(0.1-1.0 mg/kg ) | N | every 6m | height, BMI z-score | 8 |
| 8 | Effects of osmotic-release methylphenidate on height and weight in children with attention-deficit hyperactivity disorder (ADHD) following up to four years of treatment | Durá-Travé T et al | 2012 | Spain | Retrospective cohort study | osmotic-release Methylphenidate | 187(129/58) | 8.14±1.6 |  | ADHD-C:158,ADHD-I:29 | DSM-IV | Mean dose:0.89 ± 0.21 mg/kg/d after 6 months,1.31±0.2mg/kg/d after 48 months. | at least 48m | Every 6 m | BMI z-score | 5 |
| 9 | Comparative short term efficacy and tolerability of methylphenidate and atomoxetine in attention deficit hyperactivity disorder | Garg J et al | 2014 | India | Prospective Study | Immediate release | 33(27/6) | 8.47±2.22 | The tomoxetine group: 36 | ADHD-I:n=9 (27.3%), ADHD-HI:n=2 (6.1%), ADHD-C:n=22 (66.7%) | DSM-IV-TR | initial dose:5mg/d, Mean dose:0.45 mg/kg/d(range: 0.2-1 mg/kg/ d) | 8w | Every 4w | weight mean | 6 |
| 10 | Effects of methylphenidate on appetite and growth in children diagnosed with attention deficit and hyperactivity disorder | Gurbuz F et al | 2016 | Turkey | Prospective Study | prolonged release OROS-methylphenidate | 48 males | 9.8 | Healthy controls: 41 |  | DSM-IV | Fixed dose:0.5 mg/kg/d | 3m | Twice(at baseline and in the third month) | height, weight, BMI mean/z-score | 8 |
| 11 | Methylphenidate and growth in hyperactive children. A controlled withdrawal study | Klein R.G.et al | 1988 | New York | RCT |  | 58(53/5) ,ON group: 29(26/3), OFF group: 24(22/2) | 110.64m±14.2 ON group: 110m±15.0,OFF group: 111m±13.0 | OFF group: 26 |  | DSM-Ⅱ | Mean dose:41mg/d | 18.2m±7.1 | Twice(baseline and follow-up) | height, weight mean |  |
| 12 | The Effects of Methylphenidate Treatment on Child Growth in Thai Children and Adolescents with Attention-Deficit/ Hyperactivity Disorder | Koonrungsesomboon K et al | 2020 | Thai | Retrospective cohort study | immediate release: 88.1%, sustained release: 11.9% | 911(771/140) | 95.0m±19.5 (5-14) |  |  | DSM-IV-TR or DSM-5 | Mean dose: 14.1±6.2mg/d | 39.4m±23.5 (12-136m) | Every 12m | height, weight z-score | 9 |
| 13 | Psychostimulants: Influence on Body Mass Index and Height in a Pediatric Population with Attention-Deficit/Hyperactivity Disorder? | Lentferink Y E et al | 2018 | Netherlands | Retrospective cohort study | immediate release: 235 (79%), sustained release: 63 (22%) | 298(215/83) | 9.8(4.5-17.6) |  | ADHD-C:195 (65%), ADHD-I:103 (35%) UW, underweight(16);  NW, normal weigh(198);  OW, overweight(62);  obesity(22); | DSM-IV或DMS-5 | Mean dose: 0.5mg/kg/d(0.2-1.4) | 18m | Every 6m | height, BMI z-score | 5 |
| 14 | Daily methylphenidate use slows the growth of children: a community based study | Lisska M.C et al | 2003 | US (New England) | Retrospective cohort study |  | 84(68/16) | Girls:8.7±2.5 boy:8.7 ±2.7 | Siblings in the treatment group |  | DSM-IV | Mean dose: girls: 18.0±10.5 mg/d, boys: 22.5±7.8 mg/d | at lease 24m | Every 1y | BMI mean/z-score | 5 |
| 15 | Comparative effects of methylphenidate and mixed salts amphetamine on height and weight in children with attention-deficit/hyperactivity disorder | Pliszka S.R et al | 2016 | US | Retrospective cohort study | Any form | 113(94/19) | 8.5±2.1 | Mixed Salts Amphetamine group: 66 |  | DSM-IV | Mean dose:32mg/d | 2.7y±1.6 (1-7.5y) | Twice (42 patients: every 12m) | height, weightz, BMI mean | 8 |
| 16 | Effect of methylphenidate treatment on appetite and levels of leptin, ghrelin, adiponectin, and brain-derived neurotrophic factor in children and adolescents with attention deficit and hyperactivity disorder | Sahin S et al | 2014 | Turkey | Prospective Study | OROS-methylphenidate | 30(24/6) | 9.54±2.83 | Healthy controls:20 | ADHD-C:14(46.7%), ADHD-I:13(43.3%), ADHD-H:3(10%) | DSM-IV | Mean dose: 0.70±0.20 mg/kg/d | 2m | Twice(baseline and follow-up) | height, weight, BMI mean | 9 |
| 17 | Randomized controlled double-blind trial of optimal dose methylphenidate in children and adolescents with severe attention deficit hyperactivity disorder and intellectual disability | Simonoff E et al | 2013 | England | RCT | immediate release | 61(45/16) | 130m±29 | Placebo group:61 |  | ICD-10 | Three stages:  low dose (0.5mg /kg/ d),  medium dose (1.0mg /kg/ d),  and high dose (1.5mg /kg/ d) | 16w | Twice(baseline and follow-up) | weight mean |  |
| 18 | QTc prolongation after ADHD medication | Snircova E et al | 2018 | Slovakia | Prospective Study | retard capsules (sustained release capsules) | 33(27/6) | 10.6±0.5 | The tomoxetine group: 36 |  | DSM-IV-TR | initial dose:10 mg/d,increase by 10mg every week | 8w | Twice(baseline and follow-up) | BMI mean | 6 |
| 19 | ADHD in girls: Clinical comparability of a research sample | Sharp W.S et al | 1999 | US | RCT |  | 32 females | 8.9±1.7 | DEX group:32 |  | DSM-III/DSM-IV | Mean dose:0.45,0.85,and1.28mg/kg/d for weeks 1, 2, and 3 | 3w | Twice(baseline and follow-up) | weight mean |  |
| 20 | Growth deficits in children treated with desipramine: a controlled study | Spencer T et al | 1992 | US (Boston) | Retrospective cohort study |  | 29(N) | 7.8±2.4 | desipamine group,  healthy group |  | DSM-III-R | total dose:31.4±17.6mg/d,1.0±0.5 mg/kg/d | 14.2m±10.7 | Twice(baseline and follow-up) | height,weight z-score | 9 |
| 21 | Does prolonged therapy with a long-acting stimulant suppress growth in children with ADHD? | Spencer T.J et al | 2006 | US, multicenter | Prospective Study | OROS methylphenidate | 178(149/29) | 9.4±1.7 |  |  | DSM-IV | Initial dose:34.3 mg/d ->43.7 mg/d, 1.1 mg/kg/d ->1.2 mg/kg/d | ＞21m | In the first year:monthly, and then every three months | height, weight, BMI z-score | 6 |
| 22 | Does extended medication with amphetamine or methylphenidate reduce growth in hyperactive children? | Sund A.M.et al | 2002 | Norway | Retrospective cohort study |  | 23 males | 3-13 | Amphetamine group: 47 |  | ICD-10 | Mean dose:23.9±9.4mg/d | ＞12m | Yearly | height, weight mean | 6 |
| 23 | Stimulant-related reductions of growth rates in the PATS | Swanson J et al | 2006 | US, multicenter | Prospective Study | immediate release | 140(104/26) | 4.4 (preschool) |  |  | DSM-IV | Mean dose:14.2±8.1mg/d | 10m | Twice(baseline and follow-up) | height, weight mean/z-score,BMI z-score | 7 |
| 24 | ADHD and Drug Holidays: Effects on Anthropometric Changes during Methylpenidate Treatment | Turan S et al | 2021 | Turkey | Retrospective cohort study | osmotic-release, immediate-release, and pellets | 432(329/103) ADHD-C:262(197/65) ADHD-DC:170(132/38) | ADHD-C:9.78±2.74,  ADHD-DC:9.82±2.63 |  |  | DSM-IV | Mean dose:  ADHD-C group: 25.47±9.62mg/d,  ADHD-DC group: 27.36±10.67mg/d | Median:  ADHD-C: 28m;  ADHD-DC: 25m | Twice(baseline and follow-up) | height, weight, BMI z-score | 9 |
| 25 | The effects of methylphenidate on weight, height, and body mass index in Turkish children and adolescents with ADHD | Turan S et al | 2020 | Turkey | Retrospective cohort study | osmotic-release, immediate-release, and pellets | 433(330/103) | 9.81±2.72 |  | Children(6-12 y) (n=350) Adolescents (13-18 y) (n=83) | DSM-IV | Mean dose:26.18±10.08mg/d,1.35 ± 0.52 mg/kg/日 | 26m(12-113) | Twice(baseline and follow-up) | height, weight, BMI z-score | 7 |
| 26 | Growth Hormone and Thyroid Function in Children With Attention Deficit Hyperactivity Disorder Undergoing Drug Therapy | Wang L.J et al | 2022 | China (Taiwan) | Prospective Study | IR-MPH, OROS-MPH | 79(63/16) | IR-MPH: 9.0±2.3, OROS-MPH: 9.5±2.5 | None(n = 22) ATX(n = 17) |  | DSM-5 | IR-MPH:14±6.7mg/d,0.44 mg/kg/d OROS-MPH:32±9.6mg/d,0.75 mg/kg/d | 12m | Twice(baseline and follow-up) | height, BMI z-score/mean,weight mean | 9 |
| 27 | Effects of Long Acting Methylphenidate on Ghrelin Levels in Male Children with Attention Deficit Hyperactivity Disorder: An Open Label Trial | Yalcin O et al | 2014 | Turkey(Ankara) | Prospective Study | OROS methylphenidate | 33 males | 9.2±1.35 |  |  | DSM-IV | total dose:18mg/d | 2m | Twice(baseline and follow-up) | height, weight, BMI mean | 6 |
| 28 | Impact of long-term treatment of methylphenidate on height and weight of school age children with ADHD | Zhang H et al | 2010 | China | Prospective Study |  | 146(126/20) | 7.42(6.0-9.8) | non-pharmacological treatments | ADHD-HI 13 (8.9%) ADHD-I 24 (16.4%) ADHD-C 109 (74.7%) | DSM-IV | 10-20mg/d ,0.27–0.64 mg/kg/d | 2-4y | Yearly | height, weight z-score | 8 |
| 29 | Comparison of the effects of methylphenidate and the combination of methylphenidate and risperidone in preschool children with attention-deficit hyperactivity disorder | Safavi P.et al | 2016 | Iran | RCT |  | 21(18/3) | 4.52±1.24 (preschool) | Methylphenidate plus risperidone group:21 | ADHD-C:19 (90.48%), ADHD-HI:2 (9.52%) |  | Initial dose: 2.5 mg twice daily | 6w | Every 3w | weight mean |  |
| 30 | Methylphenidate and atomoxetine treatment negatively affect physical growth indexes of school-age children and adolescents with attention-deficit/hyperactivity disorder | Deng L.et al | 2021 | China | Retrospective cohort study | osmotic-release(Concerta) | 57(47/10) | 8.9±2.2 | Tomoxetine group: 29 |  | DSM-5 | Initial dose:18mg/d | 1.22y±1.01 | 0,6m | height, weight, BMI z-score | 9 |
| 31 | Effects of methylphenidate on leptin and appetite in children with attention-deficit hyperactivity disorder: an open label trial | Işeri E.et al | 2007 | Turkey | Prospective Study | Short-acting | 20 males | 9.27±1.59 | 12 age-matched healthy controls |  | DSM-IV | total dose:0.6mg/kg/d | 1m | Twice(baseline and follow-up) | height, weight, BMI mean | 6 |
| 32 | BMI Changes in Children and Adolescents with Attention Deficit Hyperactivity Disorder Before and After Treatment with Methylphenidate | Kousha M.et al | 2018 | Iran | Prospective Study |  | 90(65/25) | 9(3-18) |  |  | DSM-5 | 20 mg/d(86.9%) | 12m | Twice(baseline and follow-up) | BMI mean | 6 |
| 33 | Predictors of weight loss in children with attention deficit hyperactivity disorder treated with stimulant medication | Schertz M et al | 1996 | US | Retrospective cohort study |  | 32(29/3) | 7.5±2.1 | DEX |  | DSM-III-R | Mean dose:25.5±8.2mg/d,1.0 mg/kg/d | 11.2m±3.8 | Twice(baseline and follow-up) | height, weight z-score | 9 |

# Supplementary material 4: The quality assessment of cohort study included in this study

| Study | Representativeness of the exposed cohort | Selection of the non exposed cohort | Ascertainment of exposure | Demonstration that outcome of interest was not present at start of study | Comparability of cohorts on the basis of the design or analysis | Assessment of outcome | Was follow-up long enough for outcomes to occur | Adequacy of follow up of cohorts | Total score | Quality assessment |
| --- | --- | --- | --- | --- | --- | --- | --- | --- | --- | --- |
| Bereket A 2005 | **★** | / | **★** | **★** | / | **★** | **★** | / | 5 | Fair |
| Carucci S 2024 | **★** | **★** | **★** | **★** | **★ ★** | **★** | **★** | **★** | 9 | Good |
| Lee Y 2021 | **★** | **★** | **★** | **★** | **★** | **★** | **★** | **★** | 8 | Good |
| Çevikaslan A 2021 | **★** | / | **★** | **★** | / | **★** | **★** | / | 5 | Fair |
| Childress A C 2023 | **★** | / | **★** | **★** | / | **★** | **★** | / | 5 | Fair |
| Deng L.2021 | **★** | **★** | **★** | **★** | **★ ★** | **★** | **★** | **★** | 9 | Good |
| Díez-Suárez A 2017 | **★** | / | **★** | **★** | / | **★** | / | **★** | 5 | Fair |
| Dubnov-Raz G 2011 | **★** | **★** | **★** | **★** | **★ ★** | **★** | **★** | / | 8 | Good |
| Durá-Travé T 2012 | **★** | / | **★** | **★** | / | **★** | **★** | / | 5 | Fair |
| Garg J 2014 | **★** | / | **★** | **★** | / | **★** | **★** | **★** | 6 | Fair |
| Gurbuz F 2016 | **★** | **★** | **★** | **★** | **★** | **★** | **★** | **★** | 8 | Good |
| Işeri E.2007 | **★** | **★** | **★** | **★** | **★** | **★** | / | / | 6 | Fair |
| Koonrungsesomboon K 2020 | **★** | **★** | **★** | **★** | **★ ★** | **★** | **★** | **★** | 9 | Good |
| Lentferink Y.E. 2018 | **★** | / | **★** | **★** | / | **★** | **★** | / | 5 | Fair |
| Lisska M.C 2003 | **★** | / | **★** | **★** | / | **★** | **★** | / | 5 | Fair |
| Pliszka S.R 2016 | **★** | **★** | **★** | **★** | **★ ★** | **★** | **★** | / | 8 | Good |
| Sahin S 2014 | **★** | **★** | **★** | **★** | **★ ★** | **★** | **★** | **★** | 9 | Good |
| Schertz M et al 1996 | **★** | **★** | **★** | **★** | **★ ★** | **★** | **★** | **★** | 9 | Good |
| Snircova E 2018 | **★** | **★** | **★** | / | **★** | **★** | **★** | / | 6 | Fair |
| Spencer T 1992 | **★** | **★** | **★** | **★** | **★ ★** | **★** | **★** | **★** | 9 | Good |
| Spencer T.J 2006 | **★** | / | **★** | **★** | / | **★** | **★** | **★** | 6 | Fair |
| Sund A.M.2002 | **★** | / | **★** | **★** | / | **★** | **★** | **★** | 6 | Fair |
| Turan S 2021 | **★** | **★** | **★** | **★** | **★ ★** | **★** | **★** | **★** | 9 | Good |
| Wang L.J 2022 | **★** | **★** | **★** | **★** | **★ ★** | **★** | **★** | **★** | 9 | Good |
| Zhang H 2010 | **★** | **★** | **★** | **★** | **★** | **★** | **★** | **★** | 8 | Good |
| Kousha M.2018 | **★** | / | **★** | **★** | / | **★** | **★** | **★** | 6 | Fair |
| Turan S 2020 | **★** | / | **★** | **★** | **★** | **★** | **★** | **★** | 7 | Good |
| Yalcin O 2014 | **★** | / | **★** | **★** | / | **★** | **★** | **★** | 6 | Fair |
| Swanson J et al 2006 | **★** | / | **★** | **★** | **★** | **★** | **★** | **★** | 7 | Good |

# Supplementary material 5: GRADE assessment result

## 5.1 Height z score


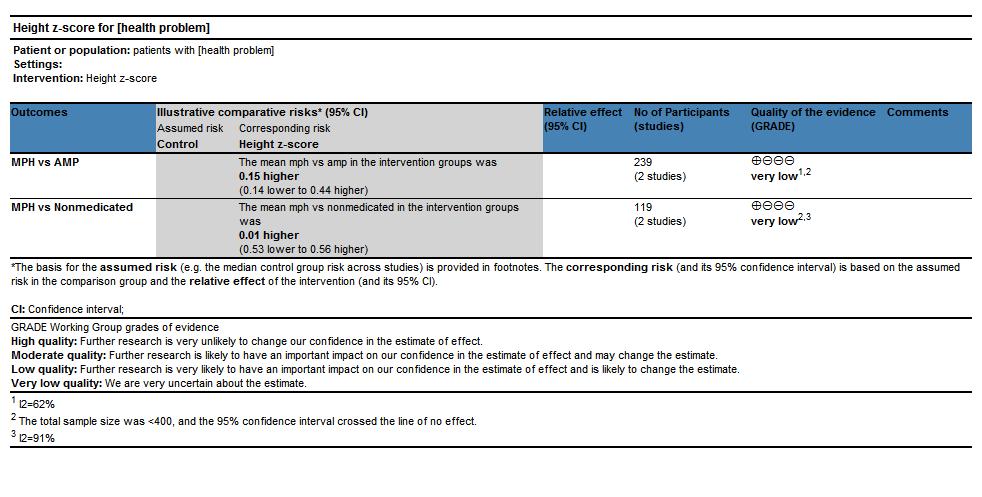


## 5.2 Weight z score


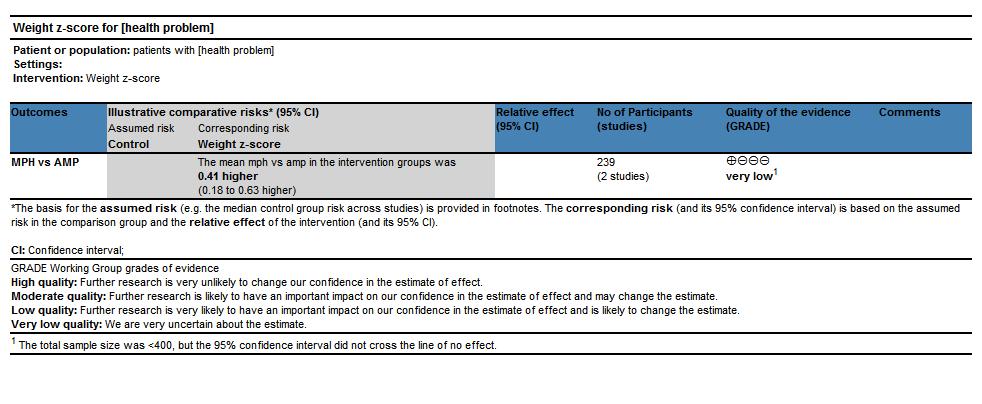


## 5.3 Mean weight


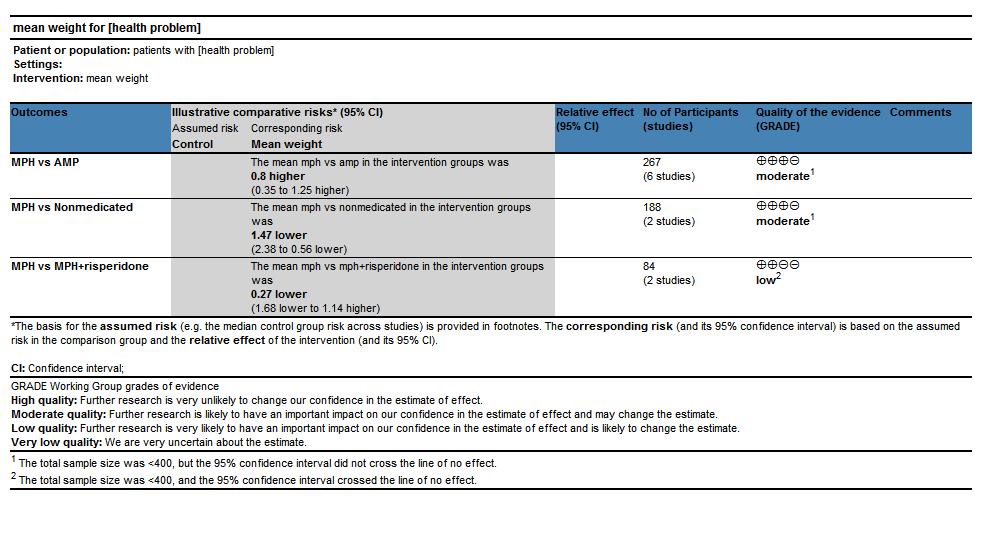


## 5.4 Mean BMI


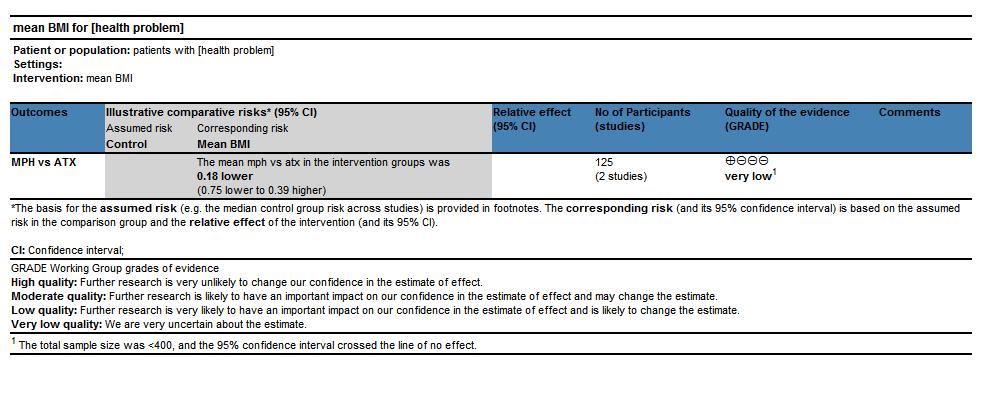


# Supplementary material 6: Results of regression analysis

## 6.1 Height z score

| mean | Coefficient | Std.err. | t | P＞\|t\| | [95% conf.interval] | |
| --- | --- | --- | --- | --- | --- | --- |
| age | 0.0020314 | 0.0221031 | 0.09 | 0.927 | -0.0427957 | 0.0468586 |
| n | -0.0000947 | 0.0001691 | -0.56 | 0.579 | -0.0004375 | 0.0002482 |
| year | -0.0007142 | 0.0042457 | -0.17 | 0.867 | -0.0093248 | 0.0078964 |

## 6.2 Weight z score

| mean | Coefficient | Std.err. | t | P＞\|t\| | [95% conf.interval] | |
| --- | --- | --- | --- | --- | --- | --- |
| age | -0.0398235 | 0.0377402 | -1.06 | 0.301 | -0.1175508 | 0.0379038 |
| n | -0.0001335 | 0.0002351 | -0.57 | 0.575 | -0.0006177 | 0.0003508 |
| year | -0.0096011 | 0.0065838 | -1.46 | 0.157 | -0.0231607 | 0.0039585 |

## 6.3 BMI z score

| mean | Coefficient | Std.err. | t | P＞\|t\| | [95% conf.interval] | |
| --- | --- | --- | --- | --- | --- | --- |
| age | -0.028931 | 0.0861745 | -0.34 | 0.739 | -0.204685 | 0.146823 |
| n | 0.0000245 | 0.0006462 | 0.04 | 0.97 | -0.0012933 | 0.0013423 |
| year | -0.0252394 | 0.0124402 | -2.03 | 0.051 | -0.0506114 | 0.0001326 |

## 6.4 Mean BMI

| mean | Coefficient | Std.err. | t | P＞\|t\| | [95% conf.interval] | |
| --- | --- | --- | --- | --- | --- | --- |
| age | 0.0911954 | 0.1557964 | 0.59 | 0.571 | -0.2559406 | 0.4383314 |
| n | 0.0053123 | 0.0083306 | 0.64 | 0.538 | -0.0132493 | 0.0238739 |
| year | 0.011854 | 0.0356184 | 0.33 | 0.746 | -0.0675088 | 0.0912168 |

# Supplementary material 7: publication bias

## 7.1 Height z score


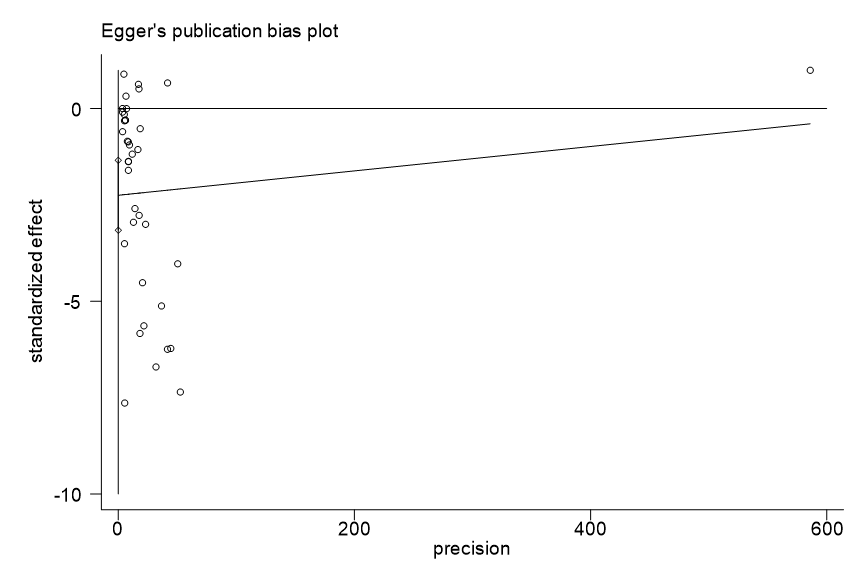


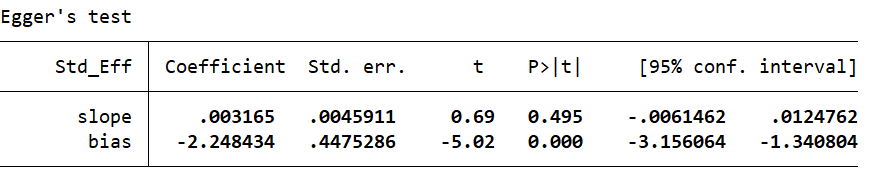


## 7.2 Weight z score


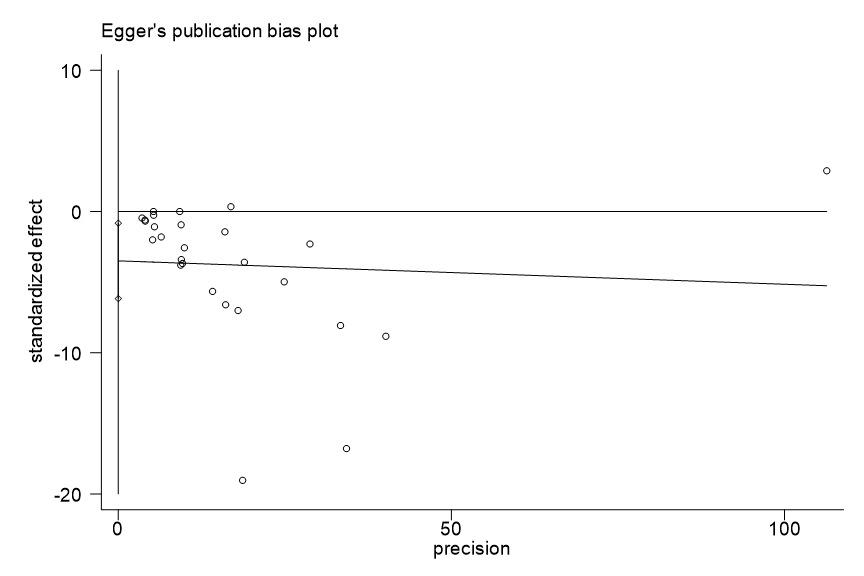

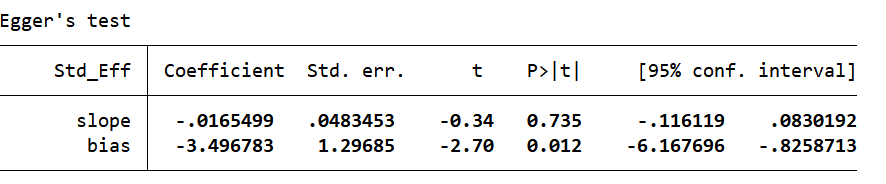


## 7.3 BMI z score


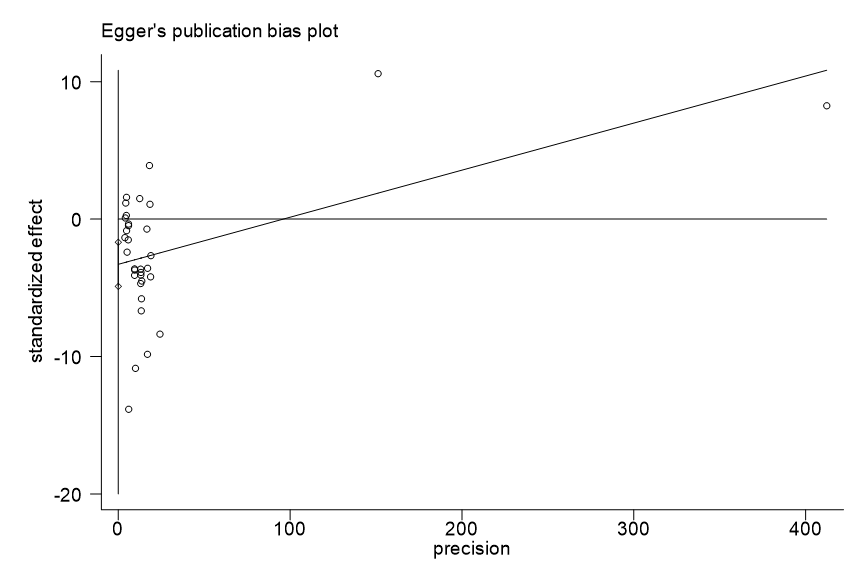

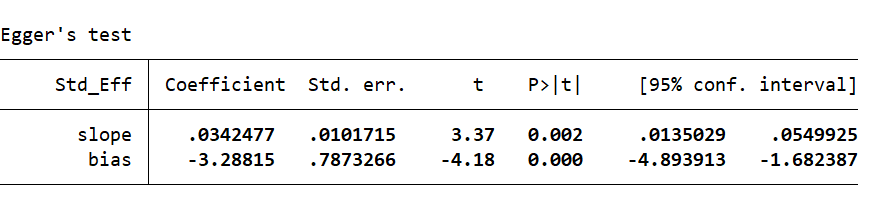


# Supplementary material 8: Trim and filling method

## 8.1 Height z score


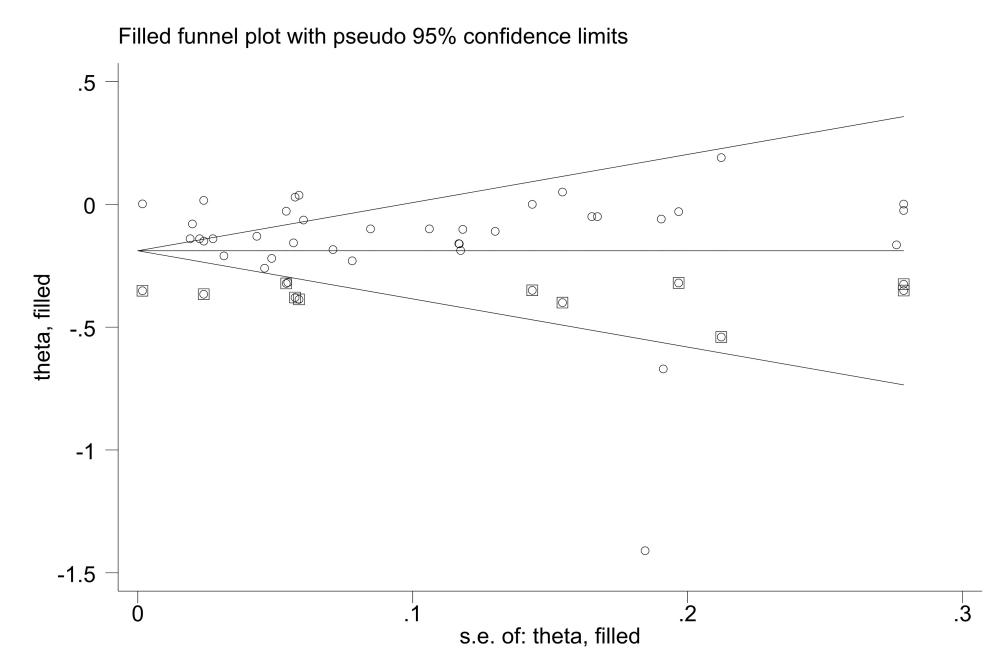


## 8.2 Weight z score


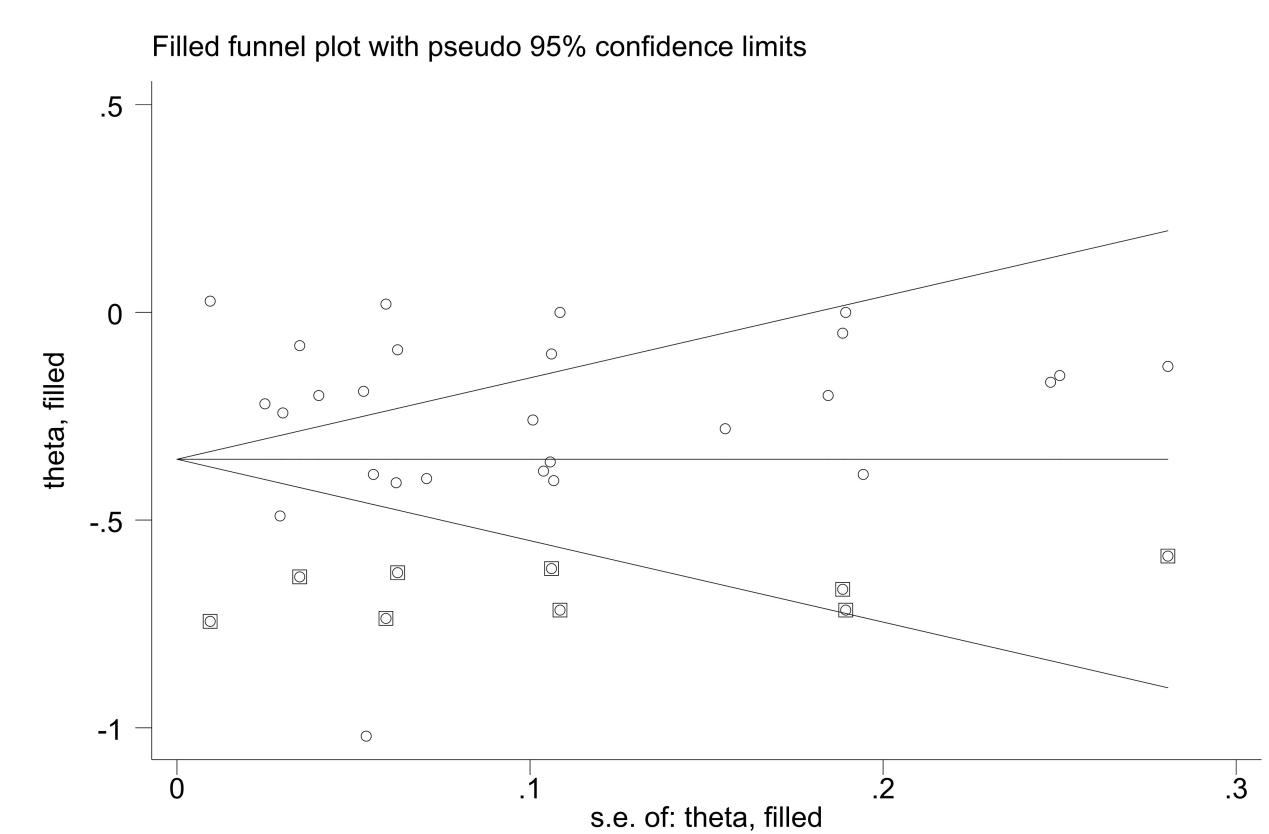


## 8.3 BMI z score


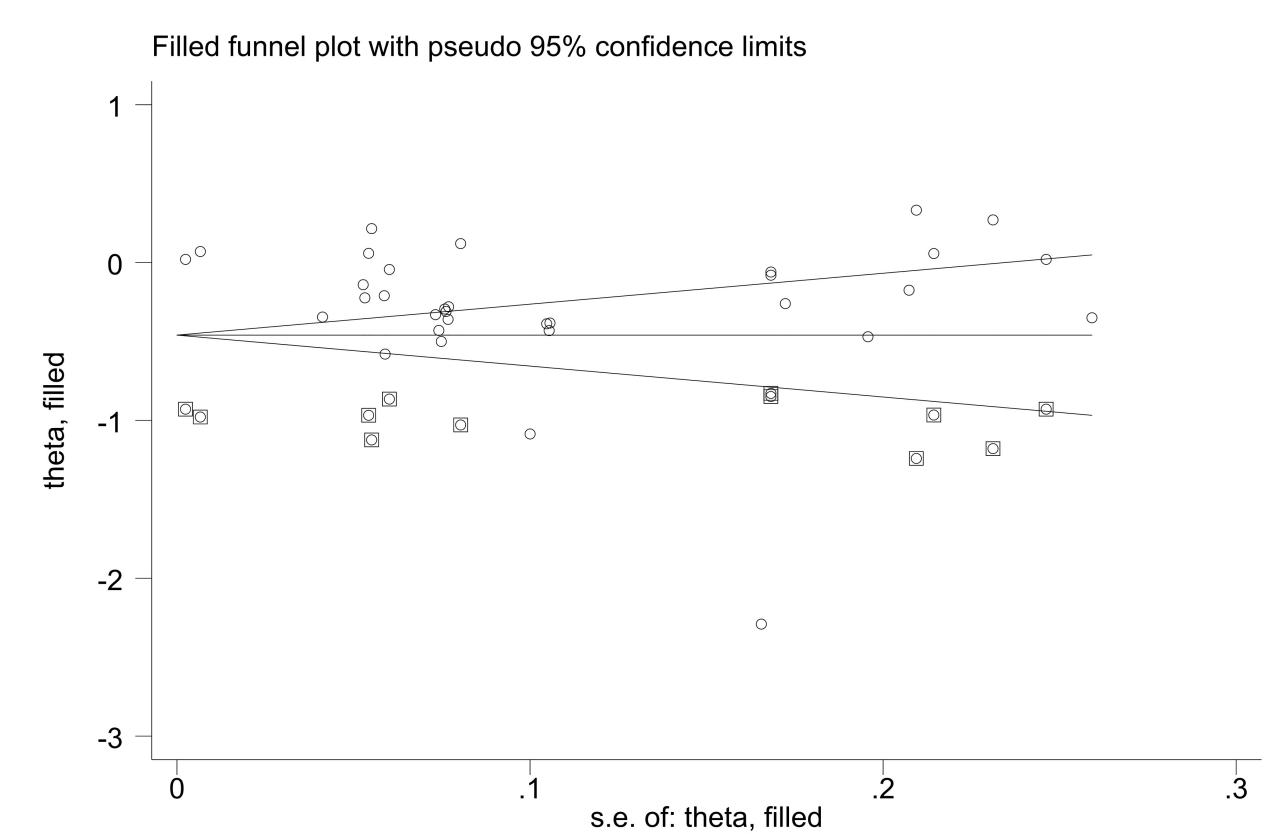

Supplement: Supplementary Material 1 — PRISMA guideline. [file SupplementaryFile1.docx]
